# Mandelonitrile lyase MDL2-mediated regulation of seed amygdalin and oil accumulation of *Prunus Sibirica*

**DOI:** 10.1186/s12870-024-05300-4

**Published:** 2024-06-21

**Authors:** Feng Chen, Junxin Zang, Zirui Wang, Jing Wang, Lingling Shi, Yu Xiu, Shanzhi Lin, Weijun Lin

**Affiliations:** 1 College of Biological Sciences and Biotechnology, National Engineering Laboratory for Tree Breeding, Key Laboratory of Genetics and Breeding in Forest Trees and Ornamental Plants, Ministry of Education, Tree and Ornamental Plant Breeding and Biotechnology Laboratory of National Forestry and Grassland Administration, Beijing Forestry University, Beijing, 100083 China; 2 West China Hospital, Sichuan University, Chengdu, 610044 Sichuan China

**Keywords:** Mandelonitrile lyase, MDL2, Amygdalin biosynthesis, Function analysis, Oil accumulation, *Prunus Sibirica* seed

## Abstract

**Background:**

The *Prunus sibirica* seeds with rich oils has great utilization, but contain amygdalin that can be hydrolyzed to release toxic HCN. Thus, how to effectively reduce seed amygdalin content of *P. sibirica* is an interesting question. Mandelonitrile is known as one key intermediate of amygdalin metabolism, but which mandelonitrile lyase (MDL) family member essential for its dissociation destined to low amygdalin accumulation in *P. sibirica* seeds still remains enigmatic. An integration of our recent 454 RNA-seq data, amygdalin and mandelonitrile content detection, qRT-PCR analysis and function determination is described as a critical attempt to determine key MDL and to highlight its function in governing mandelonitrile catabolism with low amygdalin accumulation in *Prunus sibirica* seeds for better developing edible oil and biodiesel in China.

**Results:**

To identify key MDL and to unravel its function in governing seed mandelonitrile catabolism with low amygdalin accumulation in *P. sibirica*. Global identification of mandelonitrile catabolism-associated MDLs, integrated with the across-accessions/developing stages association of accumulative amount of amygdalin and mandelonitrile with transcriptional level of *MDLs* was performed on *P*. *sibirica* seeds of 5 accessions to determine crucial MDL2 for seed mandelonitrile catabolism of *P. sibirica*. *MDL2* gene was cloned from the seeds of *P*. *sibirica*, and yeast eukaryotic expression revealed an ability of MDL2 to specifically catalyze the dissociation of mandelonitrile with the ideal values of *K*_m_ (0.22 mM) and *V*_max_ (178.57 U/mg). A combination of overexpression and mutation was conducted in Arabidopsis. Overexpression of *PsMDL2* decreased seed mandelonitrile content with an increase of oil accumulation, upregulated transcript of mandelonitrile metabolic enzymes and oil synthesis enzymes (involving FA biosynthesis and TAG assembly), but exhibited an opposite situation in *mdl2* mutant, revealing a role of PsMDL2-mediated regulation in seed amygdalin and oil biosynthesis. The *PsMDL2* gene has shown as key molecular target for bioengineering high seed oil production with low amygdalin in oilseed plants.

**Conclusions:**

This work presents the first integrated assay of genome-wide identification of mandelonitrile catabolism-related MDLs and the comparative association of transcriptional level of *MDLs* with accumulative amount of amygdalin and mandelonitrile in the seeds across different germplasms and developmental periods of *P*. *sibirica* to determine MDL2 for mandelonitrile dissociation, and an effective combination of *PsMDL2* expression and mutation, oil and mandelonitrile content detection and qRT-PCR assay was performed to unravel a mechanism of PsMDL2 for controlling amygdalin and oil production in *P*. *sibirica* seeds. These findings could offer new bioengineering strategy for high oil production with low amygdalin in oil plants.

**Supplementary Information:**

The online version contains supplementary material available at 10.1186/s12870-024-05300-4.

## Background

Cyanogenic glycosides (CNGs) are natural defense compounds identified in several plant species of different families, including economically significant fruit trees and crops (such as cassava, barley, apple, cherry, apricot, almond and wheat) [1–6]. Yet, the toxic hydrogen cyanide (HCN) is emitted from foods containing CNGs while being digested, and long-term consumption of edible plants with high level of CNGs may has a negative effect on human with adverse symptoms (such as headache, weakness, hyperventilation, vomiting, and respiratory system failure) at sub-acute cyanide dose, and even death at acute lethal dose [4, 7]. Hence, the cyanide poisoning in human has become a severe health problem in world. Several processing methods (such as peeling, fermenting, bacteria degradation, drying, boiling, soaking, and crushing) have been applied to reduce toxicity to safe level [4, 8], but all of which required a lot of time and cost, and caused serious environment pollution through release of dangerous cyanide gases or effluent water from processing factories [9–12], and thereby it is often impossible to remove all the CNGs through conventional processing. Yet, genetic engineering approach may offer an alternative method to reduce plant CNG content.

Siberian apricot (*Prunus sibirica* L.), an important fruit of Rosaceae family, is widely distributed in the mountainous areas of northeastern and northern of China, maritime territory of Russia, eastern Siberia regions, and eastern and southeastern parts of Mongolia [13]. *P. sibirica* is extensively researched in China owing to its good adaptability, rich resource, and significant economic and ecological importance [13–15]. The total area was approximately 1.7 million hectares, yielding around 192,500 metric tons of the seeds each year [14–19]. The mature dry seeds of *P. sibirica* contained a substantial amount of the oils (44.7−58.7%) with a high proportion of linoleic acid (16.4−34.7%) and oleic acid (56.2−76.3%) [13, 14, 18, 20], which has been applied in various fields including medicinal product, cosmetic, surfactant, edible oil and biodiesel feedstock [14–17, 21]. Also, the seeds show a potential usefulness in human nutrition and traditional Chinese medicine [22–26]. However, the *P. sibirica* seeds contain phenylalanine-derived amygdalin and its hydrolysis can release the HCN, causing potential toxicity and illness [13, 16], which has intercepted the commercial application of *P. sibirica* seed oils and related foods. Therefore, how to effectively reduce seed amygdalin accumulation of *P. sibirica* has become one critical concern.

Amygdalin is one of the most wide-spread groups of CNGs in the Rosaceae family, and its metabolism is generally divided into biosynthesis, bioactivation and detoxification [3, 27]. Of these, amygdalin biosynthesis is delivered from the initially synthesized mandelonitrile from Phe by two cytochrome P450 (CYP) enzymes (CYP79/CYP71), and glucosylated by UDP-glucosyltransferase (UGT1) to produce prunasin, and finally converted into amygdalin by glucosyltransferase [3, 28–30]. In bioactivation process, amygdalin is hydrolyzed to form mandelonitrile by amygdalin hydrolase (AH) and prunasin hydrolase (PH) [27, 31–33], and then hydrolyzed by mandelonitrile lyase (MDL) to form benzaldehyde with release of HCN [34]. Several plants have evolved various different detoxification mechanisms, with the most effective pathway that is the conversion of HCN into β-cyanoalanine by β-cyanoalanine synthase (β-CAS), and then hydrated by nitrilases (NITs) to generate asparagine or aspartate along with ammonia [2, 27, 35]. All these indicated that mandelonitrile was one key intermediate for both biosynthesis and bioactivation of amygdalin involved in several regulatory enzymes. Another concern was that hydroxynitrile lyases (HNLs), one kind of important enzymes in CNG-rich plants, catalyze the final step in biodegradation process of CNGs in several plants (such as cassava, Arabidopsis, Japanese apricot, peach, almond, and loquat), resulting in the productions of HCN and carbonyl component [36–44], but almost of HNLs were identified with asymmetric synthesis of mandelonitrile. Of note, MDL, one group of the HNLs, can catalyze dissociation of mandelonitrile to HCN and benzaldehyde, which was only identified in almond and black cherry [45–48]. The MDLs in *P. sibirica* seeds remain enigmatic, and thus the question of which MDL family member required for mandelonitrile dissociation destined to low seed amygdalin accumulation of *P. sibirica* has become an imperative. Recently, our 454 RNA-seq analysis of *P. sibirica* (SRX339392) [49] should ensure to effectively annotate the genes encoding MDLs responsible for mandelonitrile cleavage destined for decrease seed amygdalin amount of *P. sibirica*.

This study was designated with aim to identify crucial MDL and to highlight function in governing seed mandelonitrile catabolism with low amygdalin accumulation. To this end, we conducted a genome-wide identification of mandelonitrile catabolism-related MDLs according to recent 454 transcriptome sequencing result of *P. sibirica*, and the association of transcriptional level of all annotated *MDLs* with accumulative amount of amygdalin and mandelonitrile was performed in the seeds across different germplasms and developmental periods as first attempt to identify key MDL for mandelonitrile catabolism of *P. sibirica* seeds. The resulting *MDL2* with transcript abundance was identified, and the focus of following work was to highlight biological function of MDL2 in *P. sibirica* seeds. In this regard, *MDL2* gene was cloned from the seeds of *P*. *sibirica*, and then its heterologous expression was performed in *Pichia pastoris* to identify catalytic property. Finally, an integrated assay of *MDL2* overexpression and *mdl2* mutant was conducted in Arabidopsis, and a subsequent detection was conducted in plant growth, seed development, mandelonitrile content, oil production, and some critical regulatory enzyme transcription for mandelonitrile catabolism, oil biosynthesis [including fatty acid (FA) biosynthesis and triacylglycerol (TAG) assembly] in different transgenic lines. All these works should help to unravel MDL2-mediate regulation in seed amygdalin and oil biosynthesis of *P*. *sibirica*. The obtained results may present potential molecular target (*PsMDL2*) for future engineering high oil production with lower amygdalin in other oil plants.

## Result

### Global identification of MDL2 as crucial MDL family member for seed mandelonitrile hydrolysis of *P. sibirica*

Mandelonitrile is known as one key intermediate of amygdalin metabolism (biosynthesis and dissociation), and the less of mandelonitrile accumulation can contribute to low content of amygdalin in *P. sibirica* seeds. Thus, one vital challenge is to determine which mandelonitrile lyase (MDL) is responsible for mandelonitrile hydrolysis of *P. sibirica* seeds. In the present study, our recent 454 RNA-seq assay of *P. sibirica* (SRX339392) [49] were applied to annotate 5 genes encoding the homologies of MDL1/2/3/4/5 with differential transcriptional levels, in which *MDL2* displayed the richest transcript in *P. sibirica* seeds (Table 1), indicating that MDL2 may be as potential catabolic enzyme for seed mandelonitrile hydrolysis of *P. sibirica*.

**Table 1:** The annotated information of genes for MDLs in seeds of *Prunus sibirica* by 454 deep sequencing analysis

Unigenes-ID	Name	Definition	Location	Length	Arabidopsis ortholog	E-value	RPKM-value
comp60300_c0	MDL1	GMC oxidoreductase family protein	ER	2030	At1g72970	1E-102	6.308308
comp67704_c0	MDL2	GMC oxidoreductase family protein	ER	2144	At1g73050	3E-175	180.8585
comp34805_c0	MDL3	GMC oxidoreductase family protein	ER	2847	At1g14185	1E-125	0.112510
comp1799_c0	MDL4	GMC oxidoreductase family protein	ER	857	At1g14190	9E-101	0.082925
comp55623_c0	MDL5	GMC oxidoreductase family protein	ER	952	At1g12570	2E-131	0.084650

The data of 454 deep transcriptome sequencing of *Prunus sibirica* seeds has been deposited in NCBI/SRA database under accession number SRX339392. GMC, Glucose methanol choline; RPKM, Reads per kilobase million; ER, Endoplasmic reticulum

**Fig. 1:**
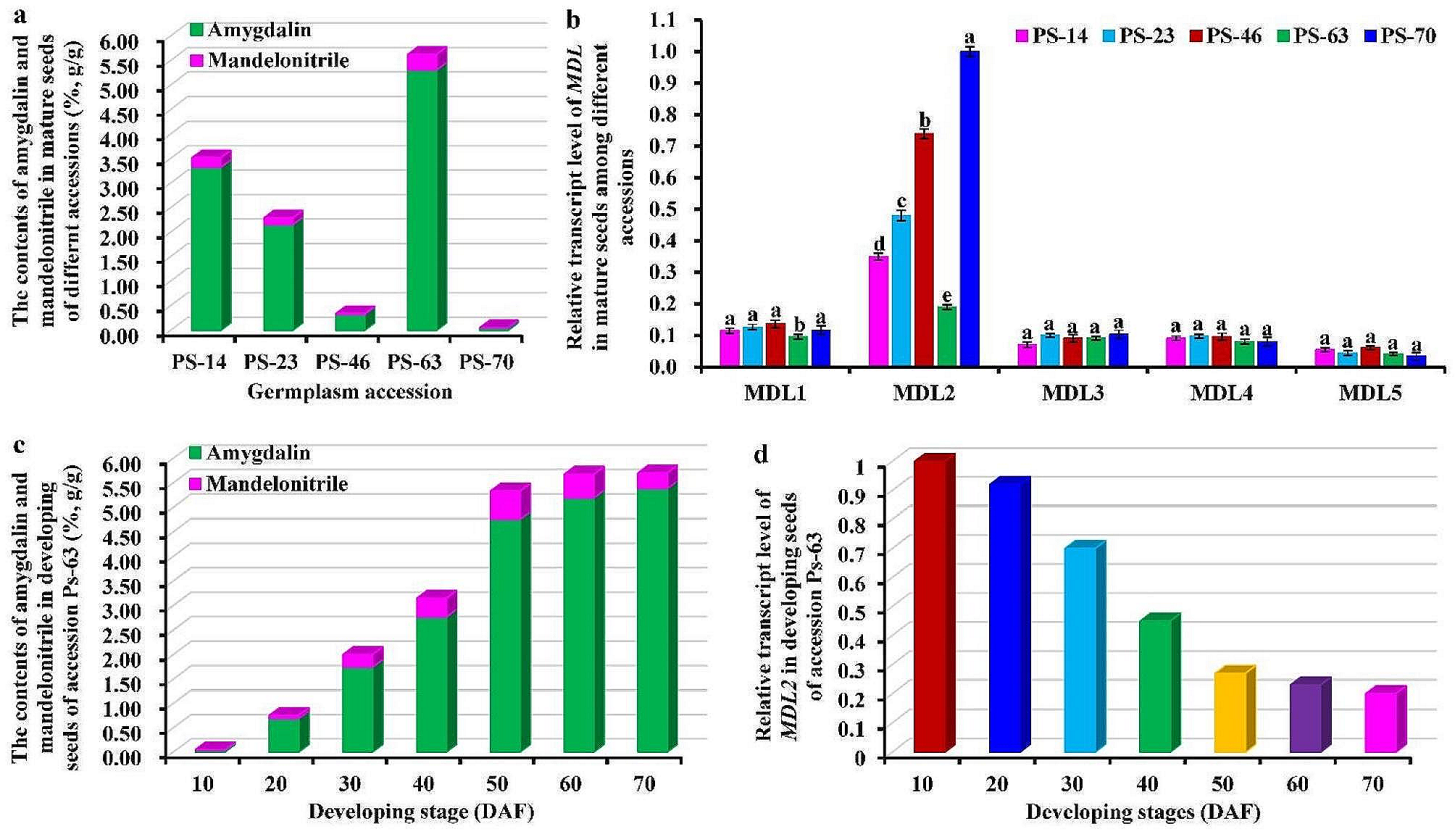
Variabilities on the contents of amygdalin and mandelonitrile and transcriptional level of ***MDL*** **gene in the seeds of different accessions or developing stages of** ***Prunus sibirica***. (**a**) Variabilities on the contents of mandelonitrile and amygdalin in mature seeds from 5 different accessions. The result was calculated and expressed as the percentage of dry seed weight (%, g/g). (**b**) Transcript analysis of our annotated MDLs in mature seeds from different accessions by qRT-PCR. The genes encoding for cyclophilin (CYP) and ubiquitin-conjugating enzyme (UBC) were used as the internal controls, and the expression level of *MDL2* in mature seeds of accession Ps-70 with the lowest seed mandelonitrile and amygdalin content was set to 1.00 for standardization. (**c**) Dynamic content changes of amygdalin and mandelonitrile in developing seeds of Ps-63 accession. The result was expressed as the percentage of dry seed weight (%, g/g). (**d**) Relative transcript level of *MDL2* in developing seeds of Ps-63 accession. The genes of *CYP* and *UBC* were used as the internal controls, and transcript level of *MDL2* in seeds at 10 DAF was set to 1.00 for standardization. All analyses were performed in three biological replicates with three technical repetitions each

To further determine MDL2 as key catabolic enzyme for mandelonitrile dissociation with low amygdalin production in *P. sibirica* seeds, five superior germplasms of *P. Sibirica* (accessions Ps-14/23/46/63/70) with high yield of the seeds were chosen as materials, and the comparative association of transcriptional level of annotated 5 *MDLs* (*MDL1/2/3/4/5*) by qRT-PCR assay with accumulative level of amygdalin and mandelonitrile was made in the seeds of different germplasms. A significant variation on the contents of mandelonitrile (0.004−0.317%) and amygdalin (0.051−5.314%) was detected in the mature seeds among different accessions. Of note, the maximum values of mandelonitrile (0.317%) and amygdalin (5.314%) were all detected for accession Ps-63, but the minimum value was recorded for Ps-70 (Fig. 1a), implying that high amygdalin accumulation may be related to high amount of mandelonitrile in the seeds across different accessions. Notably, *MDL2* transcript level was negatively correlated with the amounts of mandelonitrile and amygdalin in mature seeds of all accessions (Fig. 1a, b), while *MDL1/3/4/5* with less transcript exhibited no notable difference across different accessions (Fig. 1b). These results revealed an importance of MDL2 in regulating mandelonitrile and amygdalin accumulation of *P. sibirica* seeds, coincided with our sequencing result that *MDL2* was identified with the most abundant transcription among our all-annotated *MDLs* (Table 1).

In order to gain better insight into MDL2 in controlling mandelonitrile and amygdalin accumulation in *P. sibirica* seeds, we also explored whether the accumulative amounts of mandelonitrile and amygdalin were associated with *MDL2* transcriptional level during seed development of *P. sibirica*. One accession Ps-63 with high amygdalin was selected to analyze dynamic patterns of amygdalin and mandelonitrile contents and *MDL2* transcription in the seeds during the whole developing period from 10 DAF (unmatured stage) to 70 DAF (full ripen stage). A gradual increase in amygdalin content (0.04−5.38%) was detected in developing seeds with a rapid accumulation at 30–50 DAF (1.75−4.76%), which was the case for the content change of mandelonitrile during seed development (Fig. 1c). However, a seed development-dependently decreased pattern was detected for *MDL2* transcript in seeds during development (Fig. 1d), and notably, its transcript profile was temporally associated with the increased amount of amygdalin and mandelonitrile during seed development (Fig. 1c, d). These results indicated that the down-regulated transcript of *MDL2* may contribute likely to high accumulation of mandelonitrile and amygdalin in *P. sibirica* seeds during development. Hence, another vital challenge is to understand how MDL2 is targeted specifically to regulate mandelonitrile hydrolysis destined for low amygdalin accumulation in *P. sibirica* seeds.

### Cloning and functional assay of *PsMDL2* gene from the seeds of *P. sibirica*

**Fig. 2:**
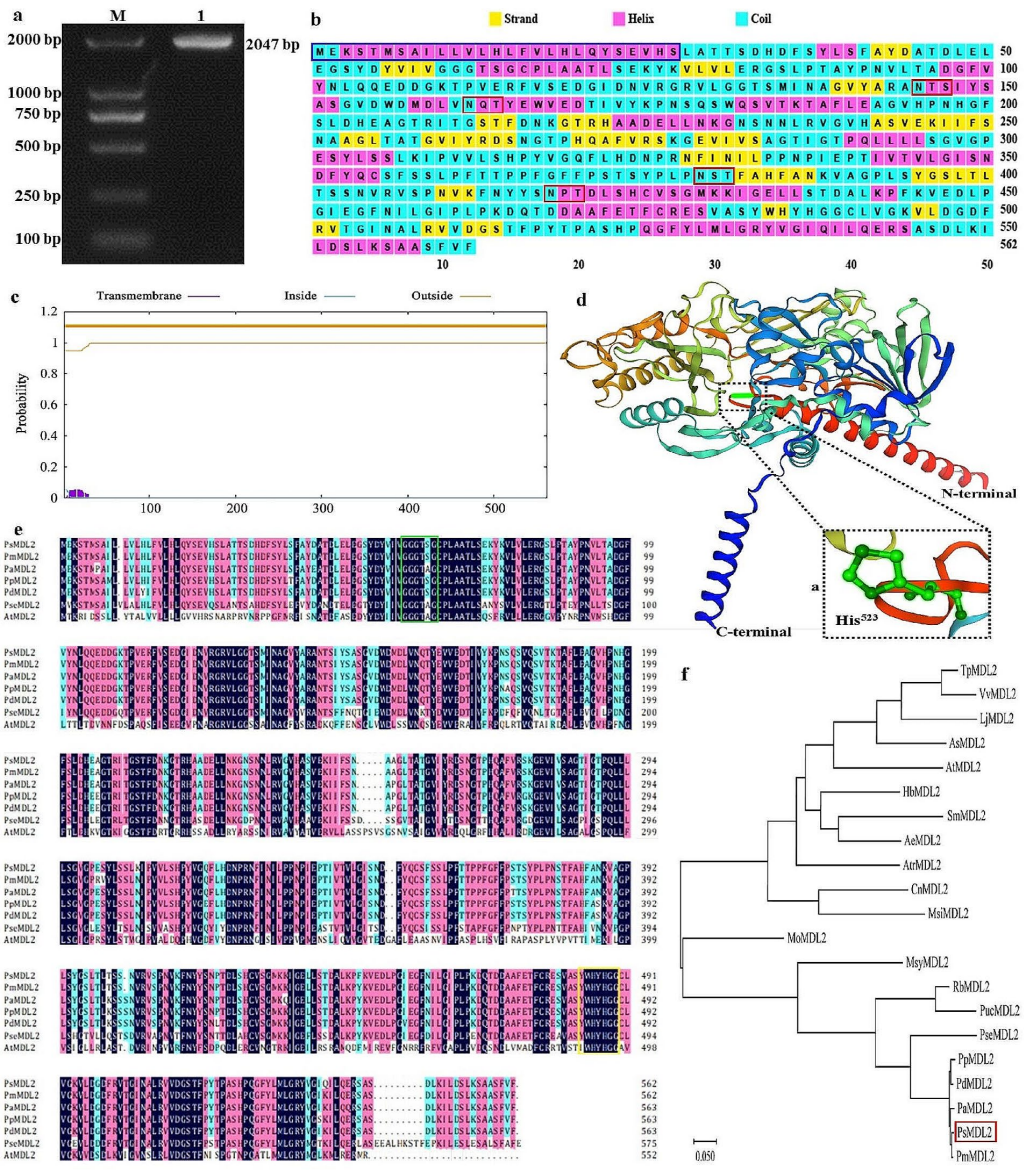
Isolation and functional analysis of ***PsMDL2*** **gene from** ***P. sibirica*seeds**. (**a**) Cloning of *PsMDL2* gene from *P. sibirica* seeds by PCR. M and line 1 signified 2000 DNA marker and *PsMDL2* gene fragment, respectively. (**b**) Assay of secondary structure for PsMDL2 by PSIPRED. The signal peptide at N-terminus was marked in blue box, and 4 potential *N*-glycosylation sites (N-X-T/S) were marked in red box. (**c**) Analysis of transmembrane domain of PsMDL2 by TMHMM. (**d**) The predicted 3D model for PsMDL2 by AlphaFold2. The active site (His) at 523 position (His^523^) of PsMDL2 protein was predicted by 3D structure model, and the purple dashed box a was a close-up view on the region of His^523^ residue, in which His^523^ was highlighted in green. (**e**) Multiple alignment assay for amino acid sequences of MDL2 proteins from *P*. *sibirica* (PsMDL2), *Prunus mume* (PmMDL2, BAO51918.1), *Prunus avium* (PaMDL2, XP_021808893.1), *Prunus persica* (PpMDL2, XP_007222890.1), *Prunus dulcis* (PdMDL2, Q945K2.1), *Prunus serotina* (PseMDL2, O50048.1), and *Arabidopsis thaliana* (AtMDL2, At1g73050). The conservative sequences (YWHYHGG) were marked in yellow box, the FAD binding motif (GGGTSG) was marked in green box, and the letters with black and other colors showed identical and similar amino acids. (**f**) Phylogenetic assay of MDL2 protein from *P. mume* (PmMDL2, BAO51918.1), *P. avium* (PaMDL2, XP_021808893.1), *P. persica* (PpMDL2, XP_007222890.1), *P. dulcis* (PdMDL2, Q945K2.1), *P. serotina* (PseMDL2, O50048.1), *Rhaphiolepis bibas* (RbMDL2, ACY69988.1), *Pyrus ussuriensis*×*Pyrus communis* (PucMDL2, KAB2624723.1), and *Malus sylvestris* (MsyMDL2, XP_050112728.1) using neighbor-joining method

In order to unravel the biological role of MDL2 in controlling mandelonitrile and amygdalin biosynthesis in the seeds of *P*. *sibirica*, we obtained 2,047-bp complete cDNA of *MDL2* (referred as *PsMDL2*) from the seeds of accession Ps-70 according to our recent 454 RNA-seq result of *P. sibirica* seeds. This cDNA sequence encodes one protein containing 562 amino acids with a molecular mass of 61.1 kD (Fig. 2a and Additional file 1: Fig. S1). PsMDL2 analysis was involved in analyzing multiple sequence alignment, constructing a phylogenetic tree, identifying conserved motif and predicting 3D protein structure. The results showed that PsMDL2 had a N-terminal signal sequence, 4 *N*-glycosylation functional motifs (N-X-T/S) (Fig. 2b), and an active-site (His residue) at the position 523 (Fig. 2d), but without transmembrane helical region (Fig. 2c). Also, PsMDL2 had a typical FAD-binding motif (residues 60 to 65, GGGTSG) and conservative sequence (YWHYHGG) (Fig. 2e), and displayed a close correlation with the MDL2 proteins from the Prunus family, including *Prunus dulcis* (PdMDL2), *P. mume* (PmMDL2), *P. avium* (PaMDL2), *P. persica* (PpMDL2), and *P. serotina* (PseMDL2) (Fig. 2e, f).

### Analysis of subcellular location and tissue-specific transcript of PsMDL2

**Fig. 3:**
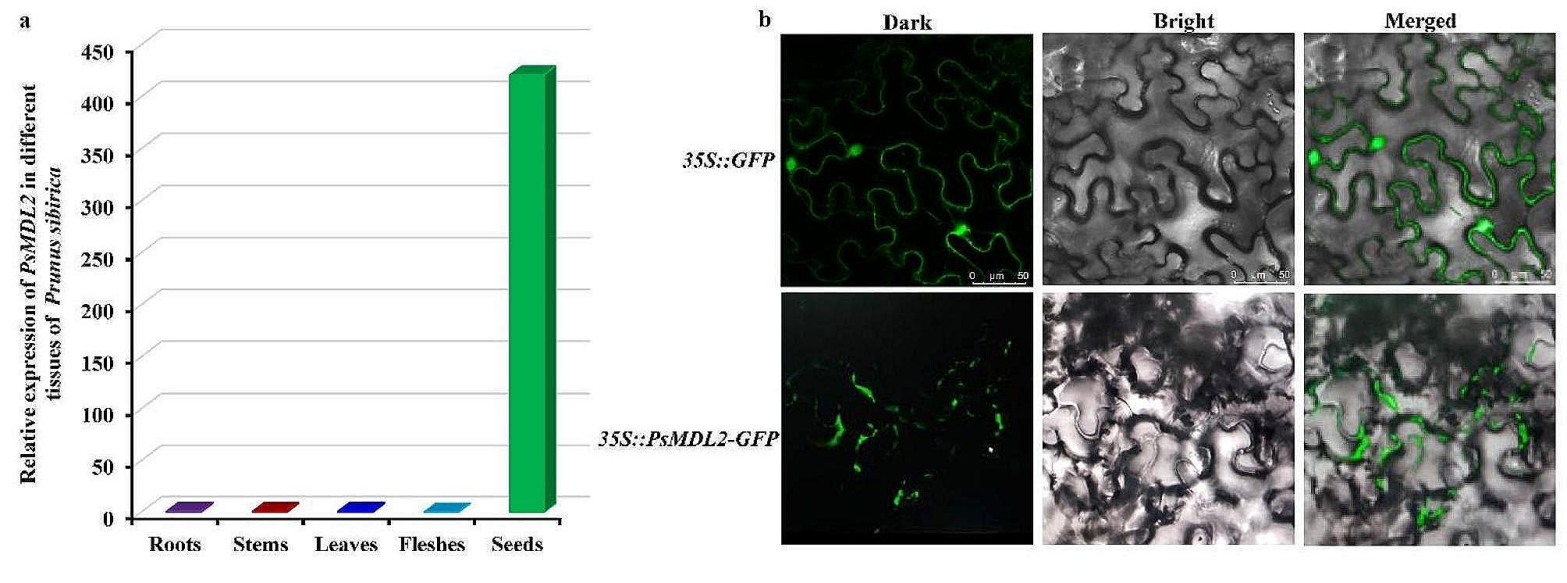
Analyses of tissue-specific expression and subcellular localization of PsMDL2. (**a**) Detection of differential transcript of *PsMDL2* in different tissues of *P*. *sibirica* by qRT-PCR. The genes encoding for cyclophilin (CYP) and ubiquitin-conjugating enzyme (UBC) was used as the inner reference, the expression level in the roots was set to 1.00 for standardization, and all the analyses were performed in three biological replicates with three technical repetitions. (**b**) Subcellular localization of PsMDL2 protein in subepidermal cells of *Nicotiana benthamiana* leaves (Bar = 50 μm). *35S::GFP* transient transformation was used as positive control. Dark was defined as the dark-field GFP-fluorescence image, bright was defined as the bright-field image, and the merged was defined as the merged image in both bright and dark fields

To explore the role of *PsMDL2* expression in governing mandelonitrile production of *P. sibirica* seeds, we examined whether *PsMDL2* gene was specifically expressed in *P. sibirica* seeds. By qRT-PCR detection, the transcriptional level of *PsMDL2* was notably increased in mature seeds (70 DAF), about 418 − 3,008 times higher than in the leaf, stem, the root or flesh (Fig. 3a), emphasizing a typical seed-specific transcript pattern of *PsMDL2* in *P. sibirica*.

Additionally, subcellular location of PsMDL2 protein was assayed using transient expression system in *N. benthamiana*. A fluorescence signal of 35S::PsMDL2-GFP fusion protein was marked within cell membrane, but the 35S::GFP control showed the fluorescence signal in cell membrane and nucleus (Fig. 3b), indicating that PsMDL2 was mainly localized in cell membrane, corresponded to the bioinformatics assay result (Fig. 2c).

### Purification of recombinant PsMDL2 from *Pichia pastoris* and detection of enzymatic property

**Fig. 4:**
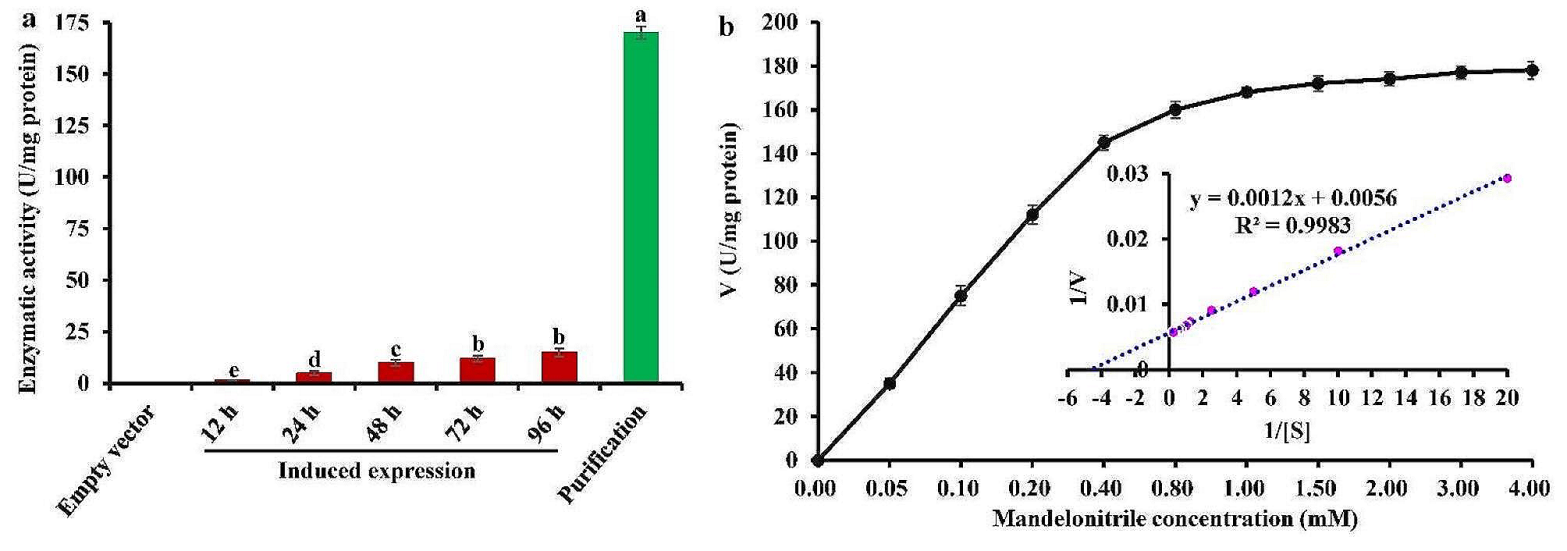
Analysis of enzymatic kinetics property of recombinant PsMDL2 by heterologous expression in ***Pichia pastoris***. (**a**) Enzymatic activity of PsMDL2 was detected for culture supernatant and purified sample of recombinant *P. pastoris* GS115. One activity unit (1U) was expressed as the amount of enzyme required for 1 µM of cleaved mandelonitrile per min under analysis condition, and the result was standardized and defined as the amount of PsMDL2 protein (U/mg protein). (**b**) Assay of enzyme kinetic characteristics of purified PsMDL2. Lineweaver-Burk plots for PsMDL2 activity in the presence of different concentrations of mandelonitrile (0.05 − 4.00 mM). l/V represented reciprocal of reaction rate, l/S represented reciprocal of the substrate (mM), *V*_max_ represented the maximum reaction rate, and *K*_m_ represented Michaelis constant. Intersection point of vertical coordinate was 1/*V*_max_, and Intersection point of abscissa coordinate was − 1/*K*_m_

To investigate the biochemical property of PsMDL2, the enzyme was heterologously expressed in yeast (*Pichia pastoris*) (Additional file 2: Fig. S2), and the recombinant enzyme was purified by a combination of ammonium sulfate and His-tag Protein Purification Kit. The PsMDL2 activity (1.51 − 14.81 U/mg) for culture supernatant of recombinant *P. pastoris* GS115 gradually increased with an increase of induction times (12–96 h) using mandelonitrile as natural substrate, and notably, enzymatic activity of purified PsMDL2 was 170.25 U/mg after 96 h of induction, about 10.5 − fold higher than that of unpurified samples, but the negative control (empty vector) showed no any catalytic activity (Fig. 4a), revealing that PsMDL2 from *P. sibirica* seeds displayed catalytic property of mandelonitrile lyase.

Both *K*_m_ and *V*_max_ are known as vital kinetic parameters of the enzyme. Here, a Lineweaver–Burk plot of recombinant PsMDL2 enzymatic activity was constructed by drawing the inverse of substrate concentration (1/[S]) against the inverse of enzyme catalytic velocity (1/v) (Fig. 4b), from which the values of *V*_max_ and *K*_m_ of PsMDL2 were calculated to be 178.57 U/mg and 0.22 mM, respectively. This further confirmed that PsMDL2 from *P. sibirica* seeds had high affinity and enzymatic activity for natural substrate (mandelonitrile).

### Expression of *PsMDL2* promotes on plant growth and seed development of transgenic lines

**Fig. 5:**
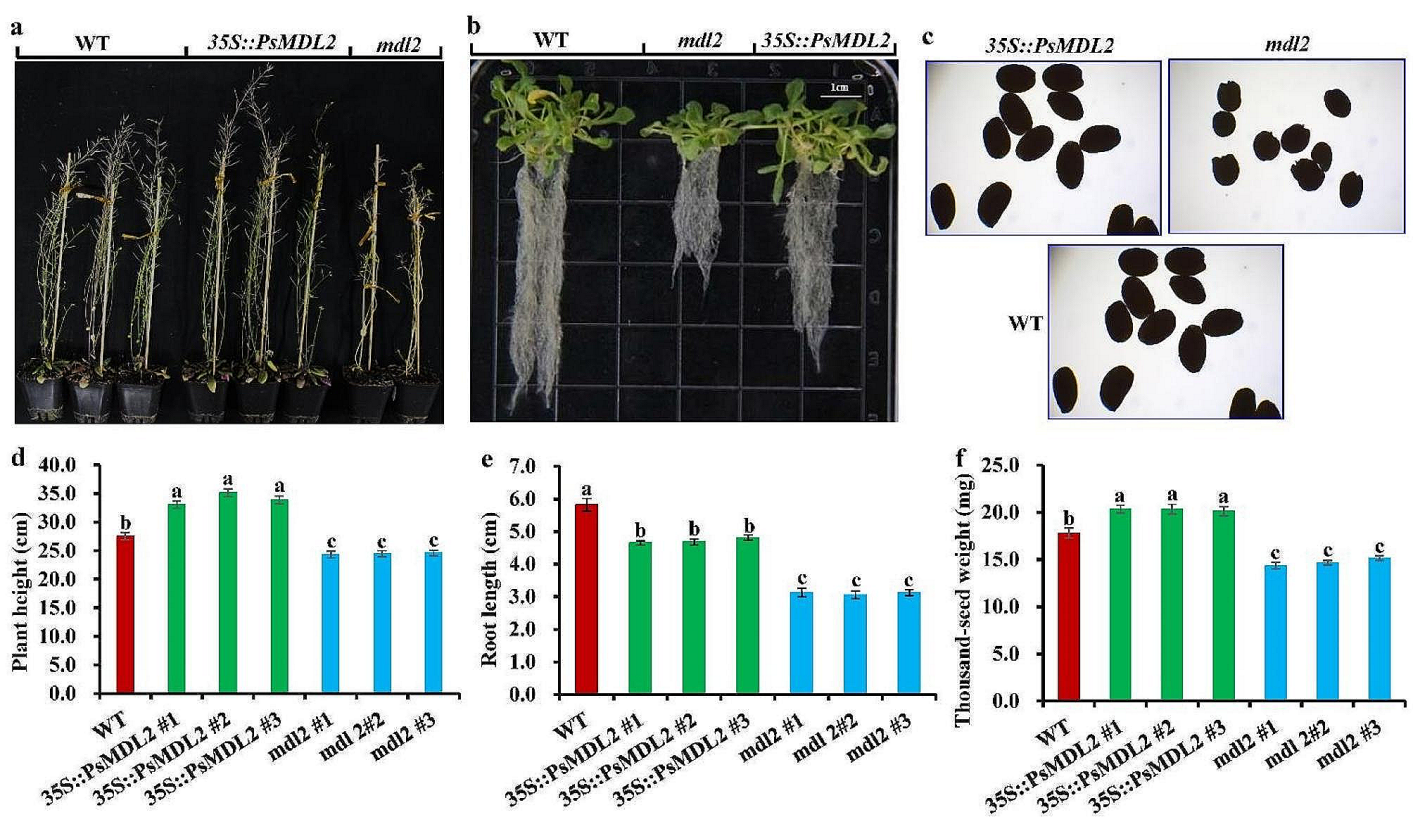
Effect of ***PsMDL2*** **expression on phenotype of transgenic Arabidopsis. (a)** Plant growth status of wild type (WT) and different transgenic Arabidopsis at 48 d after transplanting. (**b**) The root growth status of WT and different transgenic Arabidopsis seedlings at 20 d after germination. (**c**) Seed phenotype of the WT and different transgenic lines. (**d**) Analysis of plant height of different transgenic lines at 48 d after transplanting. (**e**) Analysis of root length of different transgenic lines at 20 d after germination. (**f**) Detection of thousand seed dry weight of different transgenic lines. The analyses of root length, plant height and seed weight were conducted on six biological replicates, and the results were presented as the mean ± SD, and the different letters above columns indicate significant differences at *P* < 0.05

To assess whether *PsMDL2* expression affected phenotype of transgenic plants, the traits (seed size and weight, plant height and root length) were analyzed on the seedlings from the WT (wild type, as the control), and different transgenic plants of *PsMDL2* overexpression (*35S::PsMDL2*) and its mutant (*mdl2*) from T3 homozygous seeds (Additional file 3: Table S1 and Additional file 4: Fig. S3). The plant height (33.07–35.12 cm) and thousand-seed dry weight (20.12 − 20.33 mg) of transgenic *35S::PsMDL2* lines were greater than those of the control (27.53 cm and 17.80 mg, respectively), whereas transgenic lines showed a low root length (4.65–4.81 cm) compared with the control (5.82 cm) (Fig. 5), and thereby concluded that overexpression of *PsMDL2* promoted plant growth and seed development of transgenic lines, but repressed root growth. However, plant height (24.47–25.31 cm), root length (3.05–3.13 cm) and thousand-seed dry weight (14.33 − 15.17 mg) of *mdl2* mutant plants were lesser than those of both the control and *PsMDL2* transgenic lines (Fig. 5), pointing to a negative contribution of *MDL2* mutation to plant growth and seed development.

### Expression of *PsMDL2* results in decreased mandelonitrile content of transgenic seeds

**Fig. 6:**
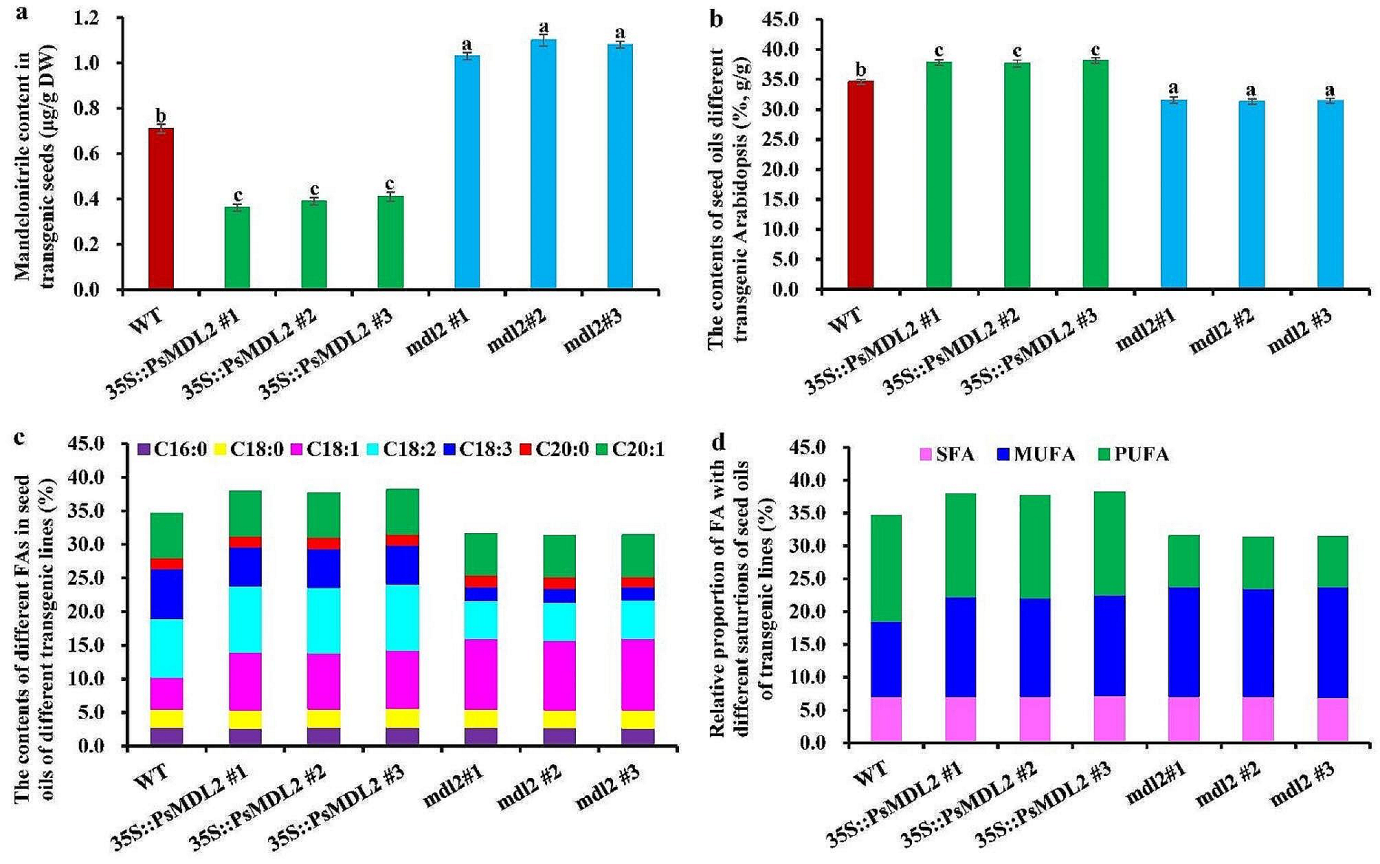
Effect of ***PsMDL2*** **expression and its mutation on the contents of mandelonitrile and oil in the seeds of different transgenic lines.** (a) Analyses of differential contents of mandelonitrile in mature seeds from different transgenic lines. (**b**) Assays of differential contents of seed oils from different transgenic lines. The content of extracted oil from each transgenic seed was calculated as the difference between the weights of seed sample before and after extraction, and the result was expressed as the percentage of the extracted oil weight to dry seed weight (%, g/g). (**c**) Detections of differential contents of various FA compositions in seed oils from different transgenic lines. The seed oils from each transgenic line were trans-esterified to detect the content of FA compositions, and the result was expressed as the percentage (%) of the obtained amount (g) of each FA methyl ester (FAME) to the amount (g) of raw seed oils. (**d**) Assays of differential levels of saturated, monounsaturated and polyunsaturated FAs in the seed oils from different transgenic lines. *35S::PsMDL2* represented overexpression of *PsMDL2* driven by CaMV35S promoter, and *mdl2* represented mutant. The results were presented as the mean ± SD by detecting three biological replicates with three technical repetitions, and the different letters above columns indicated significant difference at *P* < 0.05

To ascertain whether *PsMDL2* expression contributed functionally to seed mandelonitrile dissociation in transgenic plants, we measured mandelonitrile amount in ripen seeds of the transgenic plants (*35S::PsMDL2* and *mdl2* mutant) and control group. The mandelonitrile amount in ripen seeds of 3 independent *PsMDL2*-overexpressed lines was 0.31–0.41 µg/g DW, which was much lesser than that of the control (WT) (0.71 µg/g DW) (Fig. 6a), indicating that overexpression of *PsMDL2* could reduce seed mandelonitrile level of transgenic lines. Also consistent with this fact, *mdl2* mutant seed accumulated higher levels of mandelonitrile (1.03–1.10 µg/g DW) than both *PsMDL2* overexpressing lines and WT plants (Fig. 6a).

### Expression of *PsMDL2* increases oil accumulation of transgenic seeds with altered FA profiles

In higher plants, heterotrophic sink organs (such as seeds or fruits) are suppled carbon source mostly as sucrose, which is converted to some key precursors for oil or amygdalin biosynthesis via glycolysis and pentose phosphate pathway (PPP). This prompted us to gain insight into whether *PsMDL2* expression caused the alteration of oil content of transgenic seeds. Compared with the WT (34.61%), the increased oil content (37.66 − 38.15%) was marked in mature seeds of *35S::PsMDL2* lines, but a decrease of seed oil content (31.89 − 31.95%) was detected for *mdl2* mutants (Fig. 6b and Additional file 5: Table S2), revealing that *PsMDL2* overexpression could increase oil production of transgenic seeds.

Another question was whether *PsMDL2* expression could affect FA profiles in the seed oils of transgenic lines. Here, a total of 7 kinds of FAs were identified in the seed oils of all examined plants, including C16:0 (2.71 − 2.78%), C18:0 (2.65 − 2.79%), C18:1 (4.78 − 10.70%), C18:2 (5.68 − 9.91%), C18:3 (1.91 − 7.45%), C20:0 (1.58 − 1.69%) and C20:1 (6.11 − 6.63%) (Fig. 6c and Additional file 5: Table S2). Of these, the contents of C18:1 (8.36 − 8.70%) and C18:2 (9.83 − 9.91%) in the seed oils of *35S::PsMDL2* lines increased with a decline of C18:3 (5.71 − 5.81%) compared with the control (4.78%, 8.67% and 7.45% respectively), implying that *PsMDL2* expression could prompt production of C18:2 and C18:1 in the seed oils of transgenic plants, but suppress C18:3 synthesis. Yet, a notable increase of C18:1 (10.36 − 10.70%) was detected in seed oils of *mdl2* mutants with the decreased C18:2 (5.68 − 5.71%) and C18:3 (1.91 − 2.07%), indicating that mutation of *mdl2* could lead to 18:1 accumulation. Also, the other FAs exhibited no significant difference in seed oils among all examined seeds. These results indicated that the expression or mutation of *PsMDL2* could cause an alteration of unsaturated FAs in transgenic seed oils (Fig. 6d and Additional file 5: Table S2).

### Expression of *PsMDL2* responses specifically to seed development of transgenic plants

**Fig. 7:**
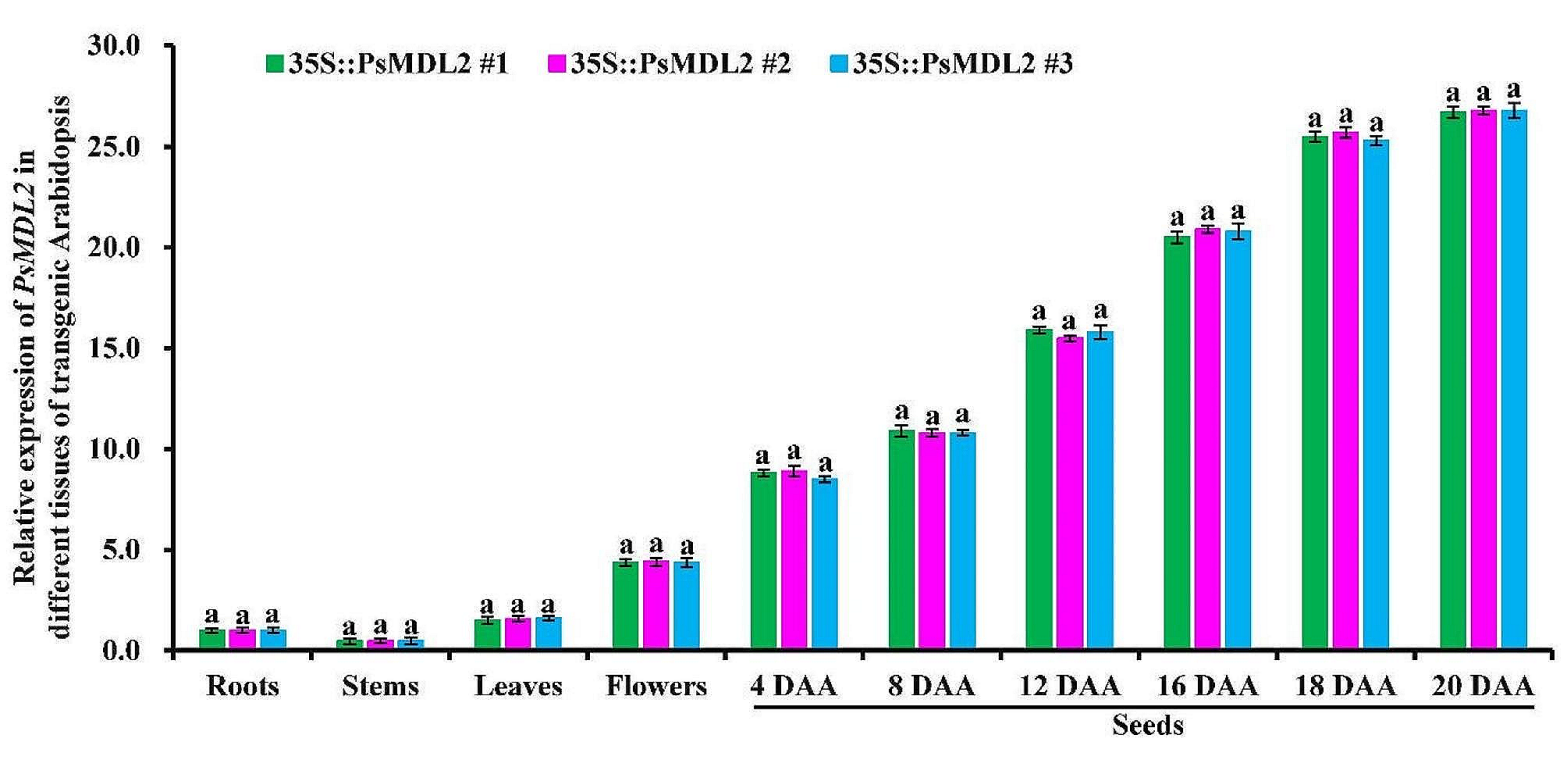
Detection of spatiotemporal expression of ***PsMDL2*** **in different tissues of transgenic Arabidopsis by qRT-PCR**. *35S::PsMDL2* represented *PsMDL2* overexpression driven by the CaMV35S promoter. The expression level from the root sample was arbitrarily set to 1.00 for standardization, and Arabidopsis *EF1α* gene was used as internal control. Error bars were SD of three biological replicates with three technical repetitions, and the different letters above columns indicated significant difference at *P* < 0.05

To highlight potential regulatory role of PsMDL2 in controlling mandelonitrile and oil accumulation of transgenic seeds, we analyzed spatiotemporal transcription pattern of *PsMDL2* in various tissues and different developing seeds of transgenic *PsMDL2* lines. A development-dependently increased transcript profile of *PsMDL2* was detected in transgenic seeds, of which a significant increase was recorded at 8–18 DAA (day after anthesis), and importantly, transcriptional amount of *PsMDL2* in developing seeds was greater than in vegetative organs (stems, roots, flowers, and leaves) (Fig. 7), and thus concluded that *PsMDL2* expression responded specifically to seed development.

### Expression of *PsMDL2* activates transcription of mandelonitrile catabolism-related genes in transgenic seeds

**Fig. 8:**
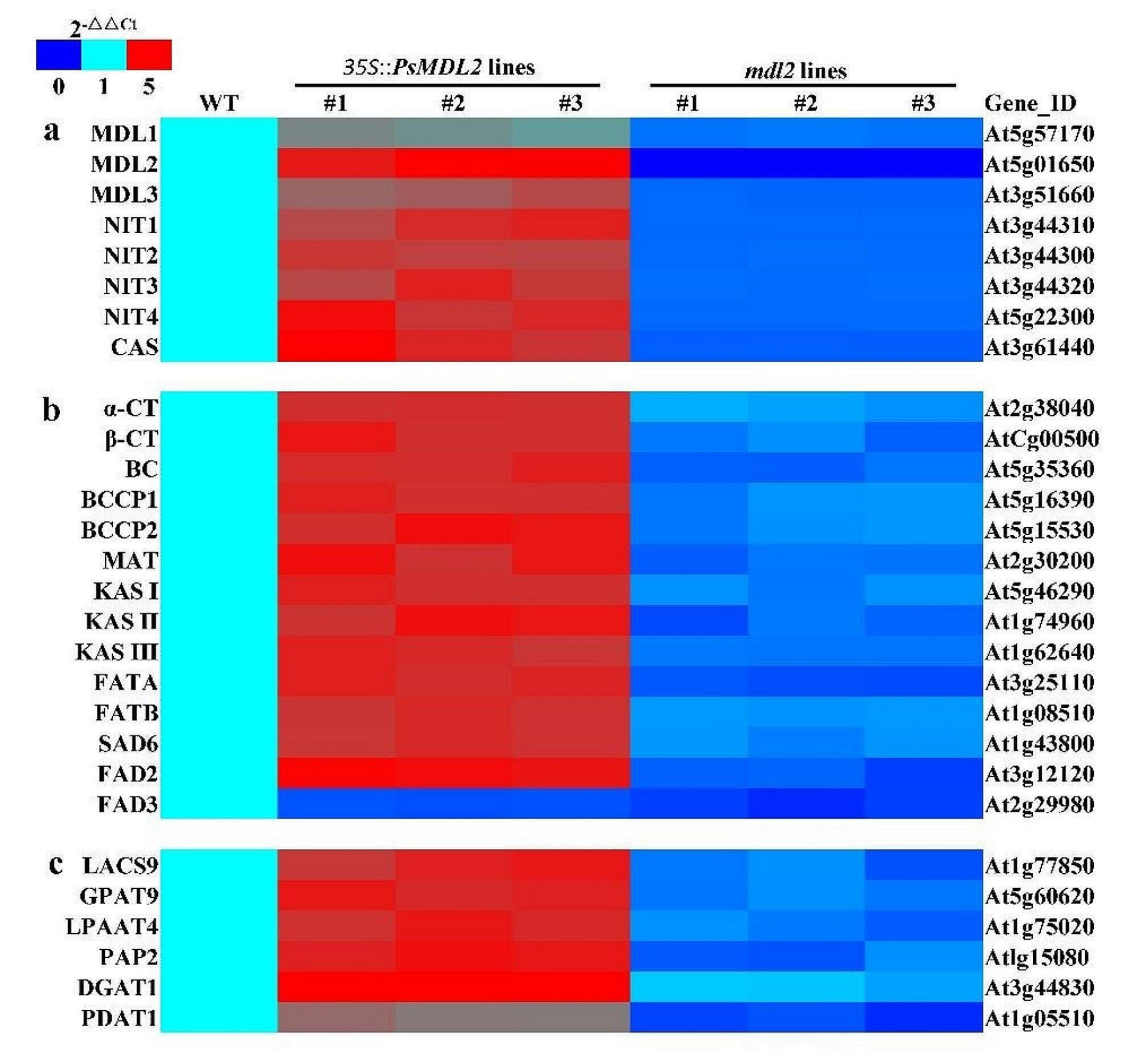
Effect of PsMDL2 on expression of the genes for the enzymes associated with mandelonitrile catabolism and oil accumulation in mature seeds of different transgenic lines of Arabidopsis by qRT-PCR. (a) Differential transcripts for mandelonitrile catabolism-related enzymes. (b) Differential transcripts for FA biosynthesis-related enzymes. (c) Differential transcripts for TAG assembly-associated enzymes. WT, *35S*::*PsMDL2* and *mdl2* represented the control, overexpression and mutant, respectively. The mature seeds (at 20 DAA) were used as the materials, and Arabidopsis *EF1α* gene was used as the internal control. The relative expression values in heatmap were counted as 2^−△△Ct^, and the expression level of the genes in mature seeds from the WT was arbitrarily set to 1.00 for standardization. Abbreviations for the enzymes are as follows: CAS, β-cyanoalanine synthase; DGAT1, acyl-CoA: DAG acyltransferase 1; EAR, enoyl-ACP reductase; FAD2/3, FA desaturases 2/3; FATA/B, fatty acyl-ACP thioesterase A/B; GPAT9, acyl-CoA: G3P acyltransferase 9; KAS I/II/III, 3-ketoacyl ACP synthase I/II/III; LACS9, long-chain acyl CoA synthetase 9; LPAAT4, acyl-CoA: LPA acyltransferase 4; MAT, malonyl-CoA-ACP transferase; MDL, mandelonitrile lyase; NIT, nitrilase; PAP2, PA phosphatase 2; PDAT, diacylglycerol (DAG) acyltransferase; SAD6, 18:0-ACP desaturase 6; TAG, triacylglycerol

To unravel molecular mechanism by which how *PsMDL2* expression was targeted specially to reduce seed mandelonitrile level of transgenic plants (Fig. 6a), we investigated whether *PsMDL2* overexpression could influence transcription of endogenous genes in relation to mandelonitrile catabolism in transgenic seeds. Thus, the ripen transgenic seeds with the maximum transcription of *PsMDL2* were chosen as materials (Fig. 7) to detect transcription of the genes for some critical enzymes of mandelonitrile catabolism [MDL1/2/3, β-cyanoalanine synthase (β-CAS) and nitrilase 1/2/3/4 (NIT1/2/3/4)] by qRT-PCR. Compared with the WT, the differentially up-regulated transcripts of endogenous *Atβ-CAS*, *AtNIT1/2/3/4* and *AtMDL1/2/3* were identified in *PsMDL2* transgenic seeds (Fig. 8a), emphasizing that *PsMDL2* overexpression effectively promoted transcription of the genes essential for mandelonitrile catabolism in transgenic seeds. Also consistent with this fact, the reduced transcription of them was marked in mature seeds of *mdl2* mutant lines (Fig. 8a). Given that the seeds of *PsMDL2* transgenic lines accumulated much lower amount of mandelonitrile than those of *mdl2* mutant lines and WT plants (Fig. 6a), it seems clear that overexpression of *PsMDL2* could lead to enhancement of mandelonitrile catabolism, destined for less accumulation of mandelonitrile in *PsMDL2* transgenic seeds.

### Expression of *PsMDL2* promotes transcription of oil synthesis-associated genes in transgenic seeds

The impact of *PsMDL2* expression on seed oils and its FA profiles of transgenic plants (Fig. 6b-d) allowed to test transcript level of endogenous genes in relation to oil accumulation in the transgenic seeds, involving in FA biosynthesis [18:0-ACP desaturase 6 (SAD6), malonyl-CoA-ACP transferase (MAT), fatty acyl-ACP thioesterase A/B (FATA/B), FA desaturase (FAD2/3), enoyl-ACP reductase (EAR), 3-ketoacyl ACP synthase I/II/III (KAS I/II/III) and acetyl-CoA carboxylase (ACC)], and TAG assembly [PA phosphatase 2 (PAP2), acyl-CoA: DAG acyltransferase 1 (DGAT1), acyl-CoA: LPA acyltransferase 4 (LPAAT4), DAG acyltransferase 1 (PDAT1), long chain acyl-CoA synthetase 9 (LACS9) and acyl-CoA: G3P acyltransferase 9 (GPAT9)], almost of which (except FAD3) showed a differential up-regulation in *PsMDL2* transgenic seeds compared with the WT, whereas the down-regulated transcript was marked in the seeds of *mdl2* mutants (Fig. 8b, c), revealing that *PsMDL2* overexpression effectively enhanced the transcriptional levels of multiple genes demand for oil biosynthesis, destined to an increase in seed oil production of transgenic plants. Another concerned was that the transcriptional level of *DGAT1* in mature seeds was significantly higher than that of *PDAT1* across all tested plants (Fig. 8b, c), reflecting that DGAT1 may be a crucial regulator for seed TAG assembly.

## Discussion

### MDL2 as crucial CYP member for seed low amygdalin accumulation of *P. sibirica* across different germplasms

Amygdalin is one of the richest groups of CNGs in the Rosaceae family (such as apricot, cherry and apple). *Prunus sibirica* (Siberian apricot), one widely distributed member of *Prunus* genus in the Rosaceae family in China, has been identified with rich seed oil content [14–20], surpassing that of other woody oilseed plants [50–54], and thus concluded that the oils from *P. sibirica* seeds had great potential as a source for edible oil and biodiesel. In this work, an obvious variation on the contents of amygdalin (0.051−5.314%) and mandelonitrile (0.004−0.317%) was detected in seeds across different accessions of *P. sibirica* (Fig. 1a), as was also noted in several *Prunus* species from different regions [55–59]. Also, both mandelonitrile and amygdalin accumulation specially responded to seed development of *P. sibirica* (Fig. 1c), which was consistent with previously published result of *P. armeniaca* kernels [57]. However, the mechanism that controlled such variation in the accumulation of amygdalin and mandelonitrile in seeds across different germplasms or developing stages remains enigmatic. In general, amygdalin is catabolized to mandelonitrile by sequential actions of amygdalin hydrolase (AH) and prunasin hydrolase (PH), and then dissociated into benzaldehyde and HCN by MDL [3, 27, 31–33]. The release of toxic HCN from amygdalin hydrolysis has become a serious health issue [16], which has greatly limited application of *P. sibirica* seed oils. Given a positive correlation of amygdalin accumulation with high content of mandelonitrile in the seeds across different germplasms or developing stages (Fig. 1a, c), it seems clear that the less of mandelonitrile biosynthesis may mostly contribute to low accumulation of amygdalin in *P. sibirica* seeds. Hence, exploration of the mechanism for controlling mandelonitrile accumulation destined to low amygdalin in seeds of *P. sibirica*s is one pivotal challenge.

Hydroxynitrile lyases (HNLs) are a group of critical enzymes in cyanogenic plants, which not only catalyze the cleavage of cyanohydrin into HCN and carbonyl compound, but also catalyze the reverse reaction for the synthesis of cyanohydrin in fine chemical or pharmaceutical industry [36, 59]. According to flavoprotein (FAD) content, HNLs is generally divided into two groups, FAD containing MDLs and non-FAD containing HNLs. Of note, almost HNLs have been identified with asymmetric synthesis of mandelonitrile in different families of cyanogenic plants [36–44], and MDLs have been reported only in black cherry and almond with specific activity for the dissociation of mandelonitrile [34, 45–48], which raised a vital question of which MDL was contributed specifically to decrease amygdalin accumulation of *P. sibirica* seeds. In this work, we applied a combined analysis of genome-wide annotation of mandelonitrile dissociation-associated MDL family members (Table 1) and the association with accumulative level of mandelonitrile and amygdalin and transcriptional amount of MDL family member in the seeds across different germplasms or developing stages of *P*. *sibirica* (Fig. 1) to determine MDL2 as critical member for low accumulation of amygdalin and mandelonitrile of *P. sibirica* seeds. Also, the PsMDL2 from the seeds of *P. sibirica* had four N-glycosylation functional sites (N-X-T/S) and one typical FAD-binding motif (GGGTSG) (Fig. 2e), as the case for other MDLs (MDL1/2/3/4/5) [46], reflecting that PsMDL2 was a FAD-dependent HNL. In support of this fact, the purified recombinant PsMDL2 displayed a specific and high activity of 170.2 U/mg for dissociation of mandelonitrile into benzaldehyde (Fig. 4a) with the *K*_m_ value (0.22 mM mandelonitrile) (Fig. 4b), which was lower than that (0.4 − 3.76 mM) reported for MDLs from *Amygdalus pedunculata*, *Eriobotrya japonica* and *Prunus armeniaca* [40, 42, 60], and thereby concluded that PsMDL2 had a high affinity of mandelonitrile. Also noteworthy was an involvement of Cys or Ser residue in catalytic mechanism of MDLs [46, 47]. Our finding that the catalytically active residue of His^523^ was identified for PsMDL2 (Fig. 2d), pointed to a diversity of MDL isoforms from different plant species.

### *PsMDL2* expression-mediated regulation for mandelonitrile and oil accumulation in of transgenic seeds

Almost FAD-independent HNLs in several plants (such as Arabidopsis, cassava, Japanese apricot, almond and peach) have been identified with asymmetric synthesis of mandelonitrile by the assay of overexpression, mutant, or catalytic activity [36–44]. To date, however, no *MDL* gene has been conducted on genetic transformation in plants to unravel its function. This work presented for the first time a combination of overexpression and mutation to identify PsMDL2 function. Given that *PsMDL2* overexpression notably decreased mandelonitrile amount in the seeds of transgenic lines, but *mdl2* mutant lines showed a great accumulation of mandelonitrile in comparison with the WT lines (Fig. 6a), it seems certain that *PsMDL2* overexpression effectively reduced mandelonitrile accumulation in transgenic seeds, which was compatible with recent report showing that mandelonitrile amount was highly increased in *hnl* mutant plants than in the WT ones with a notable reduction in *AtHNL* overexpressing lines [36]. These, combined with a negative correlation of *PsMDL2* transcriptional level with mandelonitrile accumulative amount detected in *P. sibirica* seeds across various germplasms or developmental periods (Fig. 1), revealed a key role of PsMDL2 for reduced mandelonitrile accumulation in *P. sibirica* seeds. This was in contrast to the ideal of MDL1 as crucial enzyme for the dissociation of mandelonitrile into benzaldehyde and HCN in the seeds of *P. amygdalus* and *P. serotina* [34, 46, 48]. It was also noteworthy that the plants have developed one effectively detoxicated route in which HCN can be converted by β-CAS to form β-cyanoalanine, and then hydrated by NITs to produce aspartate or asparagine and ammonia, all of which serves as reduced nitrogen source destined for plant growth [2, 3, 6]. Such pathway in our work could be clearly shown by the significantly up-regulated transcripts of endogenous *AtCAS*, *AtNIT1/2/3/4* and *AtMDL1/2/3* in the seeds of transgenic *PsMDL2* lines compared with *mdl2* mutant and the WT lines (Fig. 8a), and an obvious increase of plant height and seed weight of *PsMDL2* transgenic lines (Fig. 5), indicating that the abundantly coordinated transcriptions of them may contribute mostly to plant growth and seed development of *PsMDL2* transgenic plants. This coincided with the fact that expression of *NIT4A/NIT4B2* or *β-CAS* could promote flower and grain development [3, 5].

Another concern was the effect of *PsMDL2* overexpression or its mutation on the seed oils of transgenic plants. Given an opposite accumulation pattern between oil and mandelonitrile in the seeds across different transgenic lines (Fig. 6a, b), it seems likely that oil accumulative level might be affected by mandelonitrile amount in different transgenic seeds. It is known that the source of Phe required for phenylalanine formation destined to mandelonitrile and amygdalin synthesis is derived mostly from phenylpyruvate through glycolysis or erythrose 4-phosphate via oxidative pentose phosphate (OPP) pathway [61], both of which can also provide carbon source for acyl-CoA formation essential for FA biosynthesis and oil accumulation [14, 15, 52–54, 62–64]. Thus, the increased seed oil content in *PsMDL2*-overexpressed lines may be due to low funneling of carbon into amygdalin biosynthetic pathway. Yet, the question of how transgenic plant (overexpression or mutation) with altered amygdalin content is targeted specifically to regulate seed oil biosynthesis at the molecular level is still unknown. In oilseed plants, oil accumulation is known as a part of seed development, involving a series of gene expression. High transcripts of the enzymes required for FA biosynthesis and TAG assembly may play a key contribution to oil accumulation in the seeds of several oil plants [18, 52–54, 62, 64]. Here, the enzymes relevant for FA synthesis (EAR, KAS I/II/III, ACC, SAD6, FATA/B, MAT, and FAD2) and TAG assembly (LACS9, PAP2, GPAT9, LPAAT4, and DGAT1) were coordinated transcriptionally with PsMDL2 in the seeds of *PsMDL2* transgenic lines (Figs. 7 and 8b and c), both of which displayed a close association with the increased seed oils (Fig. 6b), but the coordinatedly down-regulated transcripts of them with low oil content were noted in the seeds of *mdl2* mutant (Figs. 6b and 8b and c), indicating that overexpression of *PsMDL2* effectively activated transcription of multiple genes crucial for oil accumulation of transgenic seeds. This conclusion could be evidenced by the results of suppression, mutant or overexpression of some key oil biosynthesis-related genes (such as *DGAT1*, *GPAT9*, *LACS4*, *FATB* and *LPAAT4*) in several oil plants [65–73].

Altogether, low mandelonitrile level with high oil amount was identified in *PsMDL2-*overexpressed seeds, but an opposite content change pattern was marked in the seeds of *mdl2* mutant, revealing that expression of *PsMDL2* would offer high seed oil production with low amygdalin content. Hence, *PsMDL2* was one key molecular target for engineering high seed oil production with low amygdalin in plants.

## Conclusions

In this work, a combined analysis of genome-wide characterization of mandelonitrile dissociation-associated MDLs and the association of mandelonitrile/amygdalin accumulative amount with transcript level of MDL family members in seeds across different germplasms or developmental periods of *P*. *sibirica* was conducted to determine MDL2 as critical family member essential for seed mandelonitrile dissociation destined to low amygdalin accumulation. *PsMDL2* showed specific and high transcription in the seeds, and had high enzymatic specificity for mandelonitrile dissociation. Overexpression of *PsMDL2* from the seeds of *P*. *sibirica* in Arabidopsis greatly promoted seed development and oil production, reduced mandelonitrile accumulation, and activated transcription of genes essential for mandelonitrile dissociation (*NIT1/2/3/4*, *CAS* and *HNL*), FA biosynthesis (*EAR*, *KAS I/II/III*, *ACC*, *SAD6*, *FATA/B*, MAT, and *FAD2*) and TAG assembly (*LACS9*, *PAP2*, *GPAT9*, *LPAAT4*, and *DGAT1*), but all of which showed an opposite situation in *mdl2* mutant. Our results revealed an importance of MDL2-mediated regulation in controlling seed amygdalin and oil biosynthesis. These results will be interest to study cyanogenic glycosides in plants, and the *PsMDL2* gene as important molecular target has the potential for bioengineering high seed oil production with low amygdalin in oilseed plant.

## Materials and methods

### Plant materials

Five superior germplasms of *P. Sibirica* (accessions Ps-14/23/46/63/70) with high-yield of the seeds were marked by our recent investigated on different *P. Sibirica* germplasms located at Beijing Changping District of China (E116°23′, N40°22′) [14], and deposited in Forest and Flower Germplasm Resource Genebank of Beijing Forestry University in China (Voucher No. 1111C0003307002308, 1111C0003307002309, 1111C0003307002347, 1111C0003307002008 and 1111C0003307002010, respectively). The different tissues (root, stem, leaf and mature fruit) were harvested from 12-year-old tree. About 150 fresh fruits with an average of 15 fruits per tree were collected respectively from 10 (unmatured period) to 70 DAF (fully ripen period) from the accessions Ps-63 and Ps-70. After the sarcocarps were removed, the plump fresh seeds were picked and kept at − 80 °C for amygdalin and mandelonitrile assay, gene cloning and qRT-PCR detection.

The seeds of wild type (WT) Arabidopsis (Col-0 ecotypes) were provided by our teams, and the seeds of Arabidopsis *mdl2* mutant (CS926813) were obtained from Nottingham Arabidopsis Stock Center (http://arabidopsis.info). For the genetic transformation of *PsMDL2*, the sterilized WT seeds were germinated on 1/2 MS medium and subjected to a 4-day cold stratification at 4 °C in the darkness. Subsequently, they were moved into one controlled chamber under specific conditions (temperature alternating between 18/22°C night and day, a photoperiod of 8/16 h night and day, and light intensity of 200 µmol m^− 2^ s^− 1^). Following a 20-day period, the plants were relocated to the pots filled with a soil blend (perlite/vermiculite/humus-soil, 1:3:3, *v*/*v*/*v*) and then cultivated under specific conditions of 8 h of darkness (22 °C) and 16 h of light (24 °C) to facilitate the genetic transformation of gene and the collection of seeds.

### Genome-wide annotation of the genes for MDL family members in the seeds of *P. sibirica*

The genes of the MDL family members were globally identified according to recent 454 RNA-seq result (SRX339392) of *P. sibirica* [49] by using BLASTX alignment against known protein databases of CDD, AP, SWISS-PROT, PFAM, TREMBL, COG, Arabidopsis proteome and NCBI nonredundant. Functional classification was analyzed by the GO terms using Blast2Go software, and KEGG pathway assignment was performed by the BLAST all against the Kyoto Encyclopedia of Genomes and Genes database.

### Detections of amygdalin and mandelonitrile in *P. sibirica* seeds

Detections of amygdalin and mandelonitrile contents were performed in mature seeds of all examined 5 germplasms and different developing seeds of two *P. sibirica* germplasms (Ps-63 and Ps-70) by HPLC. About 0.5 g of dry *P. sibirica* seeds (three samples, every germplasm or every developmental stage) was powdered and lyophilized using a freezing dryer, and then extracted for 30 min with ultrasonic generator (500 W, 40 kHz) by methanol as solute [56]. The supernatant obtained after centrifuging for at 4 °C, 10 min under 10,000 × *g* was clarified by filtration via microfiltration membrane (0.45 mm) for clarification.

HPLC analysis was conducted on Agilent 1290 Infinity II (USA) equipped with a chromatographic column (ZORBAX Eclipse Plus C18), and the temperature and pressure were respectively programmed at 40 °C and 317.0 bar. In the mobile phase of H_2_O: acetonitrile (90:10, v/v), the flow rate was 0.5 mL/min. A 10 µL of sample was injected, and the amygdalin and mandelonitrile were detected at 214 and 247 nm, respectively. The peaks were determined by comparison of known standards with their retention times, and the IBM SPSS Statistics 23 was applied to assay peak integration. The contents of amygdalin and mandelonitrile were calculated from their respective standard curves, and the obtained results were calculated as the percentage of dry seed weight (%, g/g). All analyses were presented in in three sets of biological replicates, each consisting of three technical repetitions.

### Isolation and *in silico* analyses of *PsMDL2* gene

RNA was isolated from seeds of *P. sibirica* and converted into complementary DNA using the EASYspin Plus plant RNA extraction kit and TRUEscript RT Kit (AidLab, China), respectively. Functional annotation and expression abundance were utilized to screen *PsMDL2* gene from our transcriptome database [17, 49], and the obtained sequence was applied for designing the primers by using Primer Premier 5.0 (Additional file 6: Table S3) to meet *PsMDL2* gene amplification by PCR. For the sequencing, the amplified products of *PsMDL2* were inserted into plasmid (pTOPO-TA) and then delivered into DH5α (*Escherichia coli*).

To assay open reading frame of *PsMDL2*, the Online ORFfinder (https://www.ncbi.nlm.nih.gov/orffinder/) was utilized. A maximum likelihood method was applied to construct phylogenetic tree using RAxML-NG (https://raxmlng.vital-it.ch/) [74]. Multiple sequence alignment of PsMDL2 and homologous proteins of MDL family was made by DNAMAN7.0 via ClustalX program. Subcellular location of PsMDL2 was assayed by online tools of Plant-mPLoc and BUSCA. The predictions of secondary structure and membrane spanning domain of PsMDL2 were respectively used the websites of PSIPRED 4.0 and TMHMM2.0. 3D structure of PsMDL2 was conducted by online tool AlphaFold2 and potential regulatory site for protein activity was predicted by using homologous protein MDL2 from *Prunus mume* on the UniProt website (https://www.uniprot.org/).

### Subcellular location of PsMDL2 protein

Transient expression analysis of fusion protein PsMDL2-GFP was applied to detect subcellular location of PsMDL2 in leaf cells of *Nicotiana benthamiana* [17, 18]. The complete ORF of *PsMDL2* was cloned into pBI121/*35S::GFP* to generate vector of *35S::PsMDL2*-*GFP* (Additional file 7: Fig. S4) and transformed *A. tumefaciens* GV3101 by using the freeze-thaw technique. The leaves of *N*. *benthamiana* (4-week-old) were applied for transient expression assay, and the fluorescent images were taken after 48 h of incubation at 22 °C in the dark, using positive fluorescence microscope SP8X (Leica TCS, Germany).

### Expression and purification of *PsMDL2* in *Pichia pastoris*

The complete ORF of *PsMDL2* was amplified and seamlessly cloned into *EcoR* I site of plasmid vector of pPIC9 to obtain expression vector of pPIC9/*AOX1::PsMDL2* (Additional file 6: Table S3) by Seamless Assembly Cloning Kit. The obtained construct and empty vector pPIC9/AOX1 were respectively transformed into *P. pastoris* GS115 cells, and PCR amplification was applied to verify authenticity of constructed vector. The transformant was cultured as previously described [38] with minor modification. The transformed single colony was cultivated in 50 mL of YPD medium (0.01 g/ mL yeast, 0.02 g/mL glucose, and 0.02 g/mL peptone) using a shaker under 250 rpm/min at 30 °C for 16 h, from which a portion (100.0 µL) of culture medium was introduced into 100.0 mL of BMGH medium (containing 1.0% glycerol, 0.04% biotin, and 1.34% yeast nitrogen base without amino acid). The resulting mixture was then cultured under the condition of 30 °C with a shake at 250 rpm/min to obtain the A_600_ value of 1.0 − 2.0 of the cultures. The yeast microsomes were collected after being centrifuged for 10 min at 4 °C under 4,000 × *g*, and then transferred to 100.0 mL of BMMY medium (0.5% methanol, 0.04% biotin and 1.34% yeast nitrogen base without amino acid) and cultured for 96 h at 28 °C under a shake of 250 rpm/min, during which culture medium was added with methanol at intervals of 24 h.

To effectively purify recombinant enzyme, the above culture medium was needed to undergo centrifugation at 4 °C for 10 min with a speed of 4,000 × *g*. Subsequently, the resulting supernatant was mixed with solid ammonium sulfate and continuously stirred until it reached saturation. The precipitated protein was resuspended using a buffer of sodium phosphate with 10 mM and pH 6.0 and purified with the His-tag Protein Purification Kit (Beyotime, China) after centrifuging under 10,000 × *g* for 10 min at 4 °C. To analysis enzymatic activity, the purified PsMDL2 from *P. pastoris* transformant was dissolved in 0.1 M buffer of sodium citrate (pH 5.0).

### Assay of enzymatic catalytic specificity of PsMDL2

PsMDL2 catalytic activity was tested spectrophotometrically by detecting the kinetic slope at 280 nm [39] with minor modification. The above obtained enzyme (100 µL) was added to reaction mixture (1.0 mL) of citrate-phosphate buffer (0.1 M, pH 5.0) with the substrate (mandelonitrile, 8.4 mM), and then the dynamic absorbance slope was detected at 25 °C for 5 min. The slope generated by autonomous breakdown of mandelonitrile was used as the control. One activity unit (1U) was expressed as the amount of enzyme required for 1 µM of cleaved mandelonitrile per min under the analysis condition, and the result was standardized and defined as the amount of PsMDL2 protein (U/mg protein). The content of protein was analyzed by BCA Protein Assay Kit (102,536, Abcam).

*K*_m_ (Michaelis constant) and *V*_max_ (the maximum reaction rate) as two key kinetic parameters were measured with different substrate mandelonitrile concentrations (0.05 − 4.0 mM) using Lineweaver–Burk plot of the Michaelis–Menten equation. Initial reaction rate (v) at each substrate concentration was detected, and then a Lineweaver–Burk plot of enzyme activity was made by drawing the inverse of substrate concentration (1/[S], as abscissa) against inverse of velocity of enzyme reaction (1/v, as ordinate), from which the values of *V*_max_ (U/mg protein) and *K*_m_ (mM) were calculated.

### Ectopic overexpression of *PsMDL2* in Arabidopsis

The complete ORF of *PsMDL2* was amplified and subcloned into pCAMBIA1301 directed by the promoter of CaMV35S to generate plant expression vector of pCAMBIA1301/*35S::PsMDL2*. The obtained construct vector was transformed into *A. tumefaciens* GV3101 using the freeze-thaw technique, and then delivered into WT Arabidopsis plant [75]. The T3 seeds were obtained by screening transgenic lines on 1/2MS medium (7 g/L agar, 20 g/L sucrose, and 50 mg/L kanamycin) and identifying them through PCR amplification, and then three independent-homogenous transgenic lines were obtained and examined by chi-square (*χ*^2^) test. Finally, T4 transgenic lines were applied for further analysis. All primers for constructing plant expression vector were displayed in Additional file 6: Table S3.

### Identification of Arabidopsis *mdl2* homozygous mutant

To effectively confirm T-DNA insertion (Columbia-0 background SALK_058105.13.60.X, ID: CS926813), the identifications of homozygous mutant plants of *mdl2* were performed on T3 seeds by PCR detection by the specific primers (Additional file 6: Table S3) and the *χ*^2^ test.

#### Assays of phenotypic traits of transgenic plants

To examine the impact of *PsMDL2* overexpression and its mutation on the phenotypes of transgenic plants, the traits (seed size and weight, plant height and root length) were assayed for the seedlings of T4 transgenic Arabidopsis lines (*PsMDL2* overexpression and *mdl2* mutant) and the WT. The root length was detected for the seedlings (10 days old) that were sprouted on 1/2MS medium. To detect plant height, the seedlings with 20 day old were transferred to pots and cultivated within the same chamber [53]. The weight of 1000 seeds (mg/1000 seeds) obtained from the T4 genetically modified plants and the control were determined. All determinations were conducted in triplicate.

### Detections of seed oil amount and its FA compounds

The oil content and FA compounds were detected in mature seeds of WT and T4 generation of different transgenic lines. Approximately 20 seeds were weighted and subjected to trans-methylation in 300 µL of toluene and 1 mL of methanol (including 250 µg C17:0, 50 µg BHT and 50 µL H_2_SO_4_) at a temperature of 95 °C for 1.5 h, followed by the addition of 1.5 mL sodium chloride solution (0.9%, *m*/*v*). The oils were obtained through three consecutive extractions using 1.0 mL of hexane, and evaporated under a stream of nitrogen and re-dissolved in 50 µL hexane [76]. The obtained FA methyl esters (FAMEs) was applied to analyze FA profiles using a Agilent 6890 (California, USA) gas chromatograph equipped with flame ionization detector (GC-FID) [14]. A capillary column (HP-INNOWax) with split 1:20, film thickness 0.5 μm and inner diameter 0.32 mm was employed. The temperature was firstly conducted at 60 °C and raised at a speed of 4 °C/min until reaching 220 °C. Subsequently, it was further heated to 240 °C and maintained for 10 min. Helium was used as carrier gas at a flowing speed of 1.0 mL/min. The comparison of established standards with their retention times was applied to determine the peaks of FAMEs, and the HP3398A software was applied to assay peak integration. All analyses were presented in three sets of biological replicates, each consisting of three technical repetitions.

### qRT-PCR assay

The isolated RNA from *A*. *thaliana* or *P. sibirica* by RNeasy Plant Kits (TaKaRa, Japan) was respectively quantified by a spectrophotometer (Nanodrop ND-1000, USA), and then reversely transcribed into cDNA using iScript cDNA Synthesis Kit (Bio-Rad, USA). The qRT-PCR analysis was conducted on 7500 Real-Time PCR System by using SYBR Premix Ex TaqTMII (TaKaRa, Japan), and all amplified primers used for amplification (Additional file 6: Table S3) were designed with an assistance of the PrimerQuest tool. The genes for ubiquitin-conjugating enzyme (UBC) and cyclophilin (CYP) were applied as control references [14, 15] for detecting transcript of mandelonitrile hydrolysis-related MDL family members in the seeds of *P. sibirica*. Additionally, gene expression in various tissues and different developmental seeds of transgenic plants was detected using *EF1α* gene of Arabidopsis as the control reference. For each qRT-PCR assay, three biological replicates with three technical repetitions each were performed.

### Electronic supplementary material

Below is the link to the electronic supplementary material.

Supplementary Material 1

Supplementary Material 2

Supplementary Material 3

Supplementary Material 4

Supplementary Material 5

Supplementary Material 6

Supplementary Material 7

## Data Availability

All data supporting our findings can be found in NCBI/SRA database (AccessionNo. SRX339392), and Additional files 1-7.

## Declarations

### Research involving plants

We confirm that the experimental material (*Prunus sibirica*) is owned by the authors, and deposited in the Forest and Flower Germplasm Resources Genebark of Beijing Forestry University, China.

## Abbreviations

ACC: acetyl-CoA carboxylase
AH: amygdalin hydrolase
β-CAS: β-cyanoalanine synthase
DAA: day after anthesis
DGAT1: acyl-CoA: DAG acyltransferase 1
EAR: enoyl-ACP reductase
FA: fatty acid
FAD: FA desaturase
FATA/B: fatty acyl-ACP thioesterase A/B
GPAT9: acyl-CoA: G3P acyltransferase 9
KAS I/II/III: 3-ketoacyl ACP synthase I/II/III
LACS9: long-chain acyl CoA synthetase 9
LPAAT4: acyl-CoA: LPA acyltransferase 4
MAT: malonyl-CoA-ACP transferase
MDL: mandelonitrile lyase
NIT: nitrilase
PAP2: PA phosphatase 2
PDAT: diacylglycerol (DAG) acyltransferase
PH: prunasin hydrolase
SAD6: 18:0-ACP desaturase 6
TAG: triacylglycerol

## References

[1] Lotti C, Minervini AP, Delvento C, Losciale P, Gaeta L, Sánchez-Pérez R (2023). Detection and distribution of two dominant alleles associated with the sweet kernel phenotype in almond cultivated germplasm. Front Plant Sci.

[2] Gleadow RM, Møller BL (2014). Cyanogenic glycosides: synthesis, physiology, and phenotypic plasticity. Annu Rev Plant Biol.

[3] Del Cueto J, Ionescu IA, Pičmanová M, Gericke O, Motawia MS, Olsen CE (2017). Cyanogenic glucosides and derivatives in almond and sweet cherry flower buds from dormancy to flowering. Front Plant Sci.

[4] Harenčár Ľ, Razna K, Nôžková J (2021). Cyanogenic glycosides-thier role and potential in plant food resources. J Microb Biotech Food Sci.

[5] Nielsen LJ, Stuart P, Pičmanová M, Rasmussen S, Olsen CE, Harholt J (2016). Dhurrin metabolism in the developing grain of *Sorghum bicolor* (L.) Moench investigated by metabolite profiling and novel clustering analyses of time-resolved transcriptomic data. BMC Genomics.

[6] Pičmanová M, Neilson Elizabeth H, Motawia Mohammed S, Olsen Carl E, Agerbirk N, Gray Christopher J (2015). A recycling pathway for cyanogenic glycosides evidenced by the comparative metabolic profiling in three cyanogenic plant species. Biochem J.

[7] Szczepaniak O, Stachowiak B, Jeleń H, Stuper-Szablewska K, Szambelan K, Kobus-Cisowska J (2024). The contribution of cornelian cherry (*Cornus mas* L.) alcoholic beverages on the sensory, nutritional and anti-nutritional characteristics—In vitro and in silico approaches. Processes.

[8] Bolarinwa IF, Orfila C, Morgan MRA (2014). Amygdalin content of seeds, kernels and food products commercially-available in the UK. Food Chem.

[9] Gomez MA, Berkoff KC, Gill BK, Iavarone AT, Lieberman SE, Ma JM (2023). CRISPR-Cas9-mediated knockout of *CYP79D1* and *CYP79D2* in cassava attenuates toxic cyanogen production. Front Plant Sci.

[10] Shackelford GE, Haddaway NR, Usieta HO, Pypers P, Petrovan SO, Sutherland WJ (2018). Cassava farming practices and their agricultural and environmental impacts: a systematic map protocol. Environ Evid.

[11] Montagnac JA, Davis CR, Tanumihardjo SA (2009). Processing techniques to reduce toxicity and antinutrients of cassava for use as a staple food. Compr Rev Food Sci F.

[12] Appenteng MK, Krueger R, Johnson MC, Ingold H, Bell R, Thomas AL (2021). Cyanogenic glycoside analysis in American elderberry. Molecules.

[13] Wang LB (2012). Evaluation of siberian Apricot (*Prunus Sibirica* L.) germplasm variability for biodiesel properties. J Am Oil Chem Soc.

[14] Wang J, Lin WJ, Yin ZD, Wang LB, Dong SB, An JY (2019). Comprehensive evaluation of fuel properties and complex regulation of intracellular transporters for high oil production in developing seeds of *Prunus Sibirica* for woody biodiesel. Biotechnol Biofuels.

[15] Niu J, An JY, Wang LB, Fang CL, Ha DL, Fu CY (2015). Transcriptomic analysis revealed the mechanism of oil dynamic accumulation during developing siberian apricot (*Prunus Sibirica* L.) seed kernels for the development of woody biodiesel. Biotechnol Biofuels.

[16] Liu X, Lin Z, Xiu Y, Dang Y, Lin S (2021). Analysis of the MATE family in the seeds of *Prunus Sibirica* and cloning and expression of its important member MATE40. Biotechnol Bull.

[17] Dang Y, Li W, Miao X, Xiu Y, Lin S (2022). Cloning of oleosin gene *PsOLE4* from *Prunus Sibirica* and its regulatory function analysis for oil accumulation. Biotechnol Bull.

[18] Hu J, Chen F, Zang J, Li Z, Wang J, Wang Z (2023). Native promoter-mediated transcriptional regulation of crucial oleosin protein OLE1 from *Prunus Sibirica* for seed development and high oil accumulation. Int J Biol Macromol.

[19] Wang L (2011). Resource investigation and distribute regular of three Armeniaca species. For Resour Manag.

[20] Ma Y, Wang S, Liu X, Yu H, Yu D, Li G (2021). Oil content, fatty acid composition and biodiesel properties among natural provenances of siberian apricot (*Prunus Sibirica* L.) from China. GCB Bioenergy.

[21] Wang L, Chu J (2013). Optimization of biodiesel production from siberian Apricot (*Prunus Sibirica* L.) oil using response surface methodology. Asian J Chem.

[22] Fratianni F, Ombra MN, d’Acierno A, Cipriano L, Nazzaro F (2018). Apricots: biochemistry and functional properties. Curr Opin Food Sci.

[23] Gençer A, Ozgul U, Onat SM, Gunduz G, Yaman B, Yazici H (2018). Chemical and morphological properties of Apricot wood (*Prunus armeniaca* L.) and fruit endocarp. Bartın Orman Fakültesi Derg.

[24] Akhone MA, Bains A, Tosif MM, Chawla P, Fogarasi M, Fogarasi S (2022). Apricot Kernel: Bioactivity, characterization, applications, and health attributes. Foods.

[25] Kitic D, Miladinovic B, Randjelovic M, Szopa A, Sharifi-Rad J, Calina D (2022). Anticancer potential and other pharmacological properties of *Prunus armeniaca* L.: an updated overview. Plants.

[26] Tang S, Wang M, Peng Y, Liang Y, Lei J, Tao Q et al. *Armeniacae semen amarum*: a review on its botany, phytochemistry, pharmacology, clinical application, toxicology and pharmacokinetics. Front Pharmacol. 2024;15. 10.3389/fphar.2024.1290888 PMC10844384 38323080

[27] Del Cueto J, Møller BL, Dicenta F, Sánchez-Pérez R (2018). β-Glucosidase activity in almond seeds. Plant Physiol Biochem.

[28] Franks TK, Yadollahi A, Wirthensohn MG, Guerin JR, Kaiser BN, Sedgley M (2008). A seed coat cyanohydrin glucosyltransferase is associated with bitterness in almond (*Prunus dulcis*) kernels. Funct Plant Biol.

[29] Yamaguchi T, Yamamoto K, Asano Y (2014). Identification and characterization of CYP79D16 and CYP71AN24 catalyzing the first and second steps in L-phenylalanine-derived cyanogenic glycoside biosynthesis in the Japanese apricot, *Prunus mume* Sieb. Et Zucc. Plant Mol Biol.

[30] Toledo-Martín E, García-García M, Font R, Moreno-Rojas J, Salinas-Navarro M, Gómez P (2018). Quantification of total phenolic and carotenoid content in blackberries (*Rubus Fructicosus* L.) using near infrared spectroscopy (NIRS) and multivariate analysis. Molecules.

[31] Sánchez-Pérez R, Belmonte FS, Borch J, Dicenta F, Møller BL, Jørgensen K (2012). Prunasin hydrolases during fruit development in sweet and bitter almonds. Plant Physiol Biochem.

[32] Sánchez-Pérez R, Howad W, Garcia-Mas J, Arús P, Martínez-Gómez P, Dicenta F (2010). Molecular markers for kernel bitterness in almond. Tree Genet Genom.

[33] Sánchez-Pérez R, Jørgensen K, Olsen CE, Dicenta F, Møller BL (2008). Bitterness in almonds. Plant Physiol.

[34] Suelves M, Puigdomènech P (1998). Molecular cloning of the cDNA coding for the (R)-(+)-mandelonitrile lyase of *Prunus amygdalus*: temporal and spatial expression patterns in flowers and mature seeds. Planta.

[35] Yamaguchi T (2024). Exploration and utilization of novel aldoxime, nitrile, and nitro compounds metabolizing enzymes from plants and arthropods. Biosci Biotech Biochem.

[36] Arnaiz A, Santamaria ME, Rosa-Diaz I, Garcia I, Dixit S, Vallejos S (2022). Hydroxynitrile lyase defends Arabidopsis against *Tetranychus urticae*. Plant Physiol.

[37] Siritunga D, Sayre R (2004). Engineering cyanogen synthesis and turnover in cassava (*Manihot esculenta*). Plant Mol Biol.

[38] Fukuta Y, Nanda S, Kato Y, Yurimoto H, Sakai Y, Komeda H (2011). Characterization of a new (*R*)-hydroxynitrile lyase from the Japanese apricot *Prunus mume* and cDNA cloning and secretory expression of one of the isozymes in *Pichia pastoris*. Biosci Biotechnol Biochem.

[39] Weis R, Poechlauer P, Bona R, Skranc W, Luiten R, Wubbolts M (2004). Biocatalytic conversion of unnatural substrates by recombinant almond *R*-HNL isoenzyme 5. J Mol Catal B-Enzym.

[40] Zhao G-J, Yang Z-Q, Guo Y-H (2011). Cloning and expression of hydroxynitrile lyase gene from *Eriobotrya japonica* in *Pichia pastoris*. J Biosci Bioeng.

[41] Nuylert A, Motojima F, Khanongnuch C, Hongpattarakere T, Asano Y (2020). Stabilization of hydroxynitrile lyases from two variants of passion fruit, *Passiflora edulis* Sims and *Passiflora edulis* Forma *flavicarpa* by C-terminal truncation. ChemBioChem.

[42] Yao L, Li H, Yang J, Li C, Shen Y (2018). Purification and characterization of a hydroxynitrile lyase from *Amygdalus pedunculata* pall. Int J Biol Macromol.

[43] Zheng YC, Xu JH, Wang H, Lin GQ, Hong R, Yu HL (2017). Hydroxynitrile lyase isozymes from *Prunus communis*: identification, characterization and synthetic applications. Adv Synth Catal.

[44] Nanda S, Kato Y, Asano Y (2005). A new (*R*)-hydroxynitrile lyase from *Prunus mume*: asymmetric synthesis of cyanohydrins. Tetrahedron.

[45] Hu Z, Poulton JE (1997). Sequencing, genomic organization, and preliminary promoter analysis of a black cherry (*R*)-(+)-mandelonitrile lyase gene. Plant Physiol.

[46] Hu Z, Poulton JE (1999). Molecular analysis of (*R*)-(+)-mandelonitrile lyase microheterogeneity in black cherry. Plant Physiol.

[47] Wajant H, Forster S, Selmar D, Effenberger F, Pfizenmaier K (1995). Purification and characterization of a novel (*R*)-mandelonitrile lyase from the fern *Phlebodium aureum*. Plant Physiol.

[48] Zheng L, Poulton JE (1995). Temporal and spatial expression of amygdalin hydrolase and (*R*)-(+)-mandelonitrile lyase in black cherry seeds. Plant Physiol.

[49] Dong SB, Liu YL, Niu J, Ning Y, Lin SZ, Zhang ZX (2014). *De novo* transcriptome analysis of the siberian apricot (*Prunus Sibirica* L.) and search for potential SSR markers by 454 pyrosequencing. Gene.

[50] Guo JY, Li HY, Fan SQ, Liang TY, Yu HY, Li JR (2015). Genetic variability of biodiesel properties in some *Prunus* L.(Rosaceae) species collected from Inner Mongolia, China. Ind Crop Prod.

[51] Wang LB (2013). Properties of Manchurian apricot (*Prunus Mandshurica* Skv.) and Siberian apricot (*Prunus Sibirica* L.) seed kernel oils and evaluation as biodiesel feedstocks. Ind Crop Prod.

[52] Lin Z, Chen F, Wang H, Hu J, Shi L, Zhang Z (2023). Evaluation of oil accumulation and biodiesel property of *Lindera Glauca* fruits among different germplasms and revelation of high oil producing mechanism for developing biodiesel. Biotechnol Biof Biop.

[53] Chen F, Lin W, Li W, Hu J, Li Z, Shi L (2023). Determination of superior *Pistacia chinensis* accession with high-quality seed oil and biodiesel production and revelation of LEC1/WRI1-mediated high oil accumulative mechanism for better developing woody biodiesel. BMC Plant Biol.

[54] Lin Z, An J, Wang J, Niu J, Ma C, Wang L (2017). Integrated analysis of 454 and Illumina transcriptomic sequencing characterizes carbon flux and energy source for fatty acid synthesis in developing *Lindera Glauca* fruits for woody biodiesel. Biotechnol Biofuels.

[55] Lehmane H, Kohonou AN, Tchogou AP, Ba R, Dah-Nouvlessounon D, Didagbé O (2023). Antioxidant, anti-inflammatory, and anti-cancer properties of amygdalin extracted from three cassava varieties cultivated in Benin. Molecules.

[56] Wang W, Xiao X-Z, Xu X-Q, Li Z-J, Zhang J-M (2022). Variation in amygdalin content in kernels of six almond species (*Prunus* Spp. L.) distributed in China. Front Plant Sci.

[57] Deng P, Cui B, Zhu H, Phommakoun B, Zhang D, Li Y (2021). Accumulation pattern of amygdalin and prunasin and its correlation with fruit and kernel agronomic characteristics during Apricot (*Prunus armeniaca* L.) kernel development. Foods.

[58] Karsavuran N, Charehsaz M, Celik H, Asma BM, Yakıncı C, Aydın A (2014). Amygdalin in bitter and sweet seeds of apricots. Toxicol Environ Chem.

[59] Bhalla TC, Kumar V, Kumar V, Thakur N (2018). Savitri n. Nitrile metabolizing enzymes in biocatalysis and biotransformation. Appl Biochem Biotechnol.

[60] Asif M, Bhalla TC (2016). Hydroxynitrile lyase of wild apricot (*Prunus armeniaca* L.): purification, characterization and application in synthesis of enantiopure mandelonitrile. Catal Lett.

[61] Galili G, Amir R, Fernie AR (2016). The regulation of essential amino acid synthesis and accumulation in plants. Annu Rev Plant Biol.

[62] Bourgis F, Kilaru A, Cao X, Ngando-Ebongue G-F, Drira N, Ohlrogge JB (2011). Comparative transcriptome and metabolite analysis of oil palm and date palm mesocarp that differ dramatically in carbon partitioning. Proc Natl Acad Sci USA.

[63] Bates PD, Stymne S, Ohlrogge J (2013). Biochemical pathways in seed oil synthesis. Curr Opin Plant Biol.

[64] Schwender J, Hebbelmann I, Heinzel N, Hildebrandt T, Rogers A, Naik D (2015). Quantitative multilevel analysis of central metabolism in developing oilseeds of oilseed rape during in vitro culture. Plant Physiol.

[65] Zhang TT, He H, Xu CJ, Fu Q, Tao YB, Xu R (2021). Overexpression of type 1 and 2 diacylglycerol acyltransferase genes (*JcDGAT1* and *JcDGAT2*) enhances oil production in the woody perennial biofuel plant *Jatropha curcas*. Plants.

[66] Misra A, Khan K, Niranjan A, Nath P, Sane VA (2013). Over-expression of JcDGAT1 from *Jatropha curcas* increases seed oil levels and alters oil quality in transgenic *Arabidopsis thaliana*. Phytochemistry.

[67] Maravi DK, Kumar S, Sharma PK, Kobayashi Y, Goud VV, Sakurai N (2016). Ectopic expression of *AtDGAT1*, encoding diacylglycerol *O*-acyltransferase exclusively committed to TAG biosynthesis, enhances oil accumulation in seeds and leaves of Jatropha. Biotechnol Biofuels.

[68] Torabi S, Sukumaran A, Dhaubhadel S, Johnson SE, LaFayette P, Parrott WA (2021). Effects of type I *diacylglycerol O-acyltransferase* (*DGAT1*) genes on soybean (*Glycine max* L.) seed composition. Sci Rep.

[69] Regmi A, Shockey J, Kotapati HK, Bates PD (2020). Oil-producing metabolons containing DGAT1 use separate substrate pools from those containing DGAT2 or PDAT. Plant Physiol.

[70] Zhao J, Bi R, Li S, Zhou D, Bai Y, Jing G (2019). Genome-wide analysis and functional characterization of Acyl-CoA:diacylglycerol acyltransferase from soybean identify *GmDGAT1A* and *1B* roles in oil synthesis in Arabidopsis seeds. J Plant Physiol.

[71] Zhang M, Fan J, Taylor DC, Ohlrogge JB (2009). *DGAT1* and *PDAT1* acyltransferases have overlapping functions in *Arabidopsis* triacylglycerol biosynthesis and are essential for normal pollen and seed development. Plant Cell.

[72] Liu F, Zhao Y-P, Zhu H-g, Zhu Q-H, Sun J (2017). Simultaneous silencing of *GhFAD2-1* and *GhFATB* enhances the quality of cottonseed oil with high oleic acid. J Plant Physiol.

[73] Zhang Q, Yu R, Sun D, Bai Z, Li H, Xue L (2017). PrLPAAT4, a putative lysophosphatidic acid acyltransferase from *Paeonia rockii*, plays an important role in seed fatty acid biosynthesis. Molecules.

[74] Kozlov AM, Darriba D, Flouri T, Morel B, Stamatakis A (2019). RAxML-NG: a fast, scalable and user-friendly tool for maximum likelihood phylogenetic inference. Bioinformatics.

[75] Clough SJ, Bent AF (1998). Floral dip: a simplified method for *Agrobacterium*-mediated transformation of *Arabidopsis thaliana*. Plant J.

[76] Li Y, Beisson F, Pollard M, Ohlrogge J (2006). Oil content of Arabidopsis seeds: the influence of seed anatomy, light and plant-to-plant variation. Phytochemistry.

