# Supplementary material for: Mandelonitrile lyase MDL2-mediated regulation of seed amygdalin and oil accumulation of Prunus Sibirica
Source: BMC Plant Biol. 2024 Jun 21;24:590. doi: 10.1186/s12870-024-05300-4 (PMC11191352; doi:10.1186/s12870-024-05300-4)
Supplement: Supplementary file 1 — Supplementary Material 1 [file 12870_2024_5300_MOESM1_ESM.docx]

**
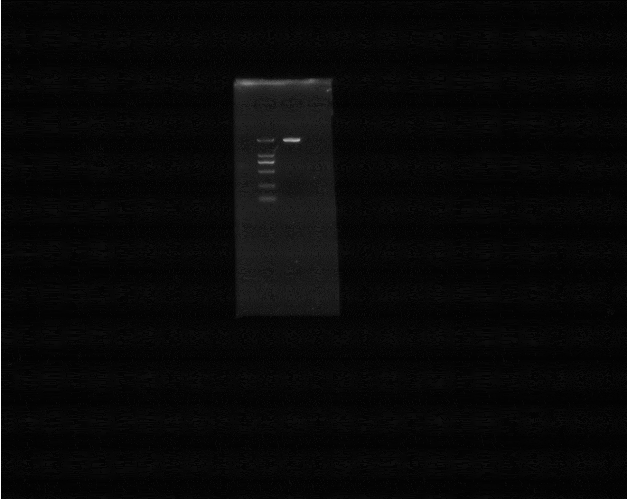
**

**Figure S1. The original** **full-length gel image matched to the cropped version in Figure 2a of the manuscript**.
