# Supplementary material for: Mandelonitrile lyase MDL2-mediated regulation of seed amygdalin and oil accumulation of Prunus Sibirica
Source: BMC Plant Biol. 2024 Jun 21;24:590. doi: 10.1186/s12870-024-05300-4 (PMC11191352; doi:10.1186/s12870-024-05300-4)
Supplement: Supplementary file 2 — Supplementary Material 2 [file 12870_2024_5300_MOESM2_ESM.docx]

**
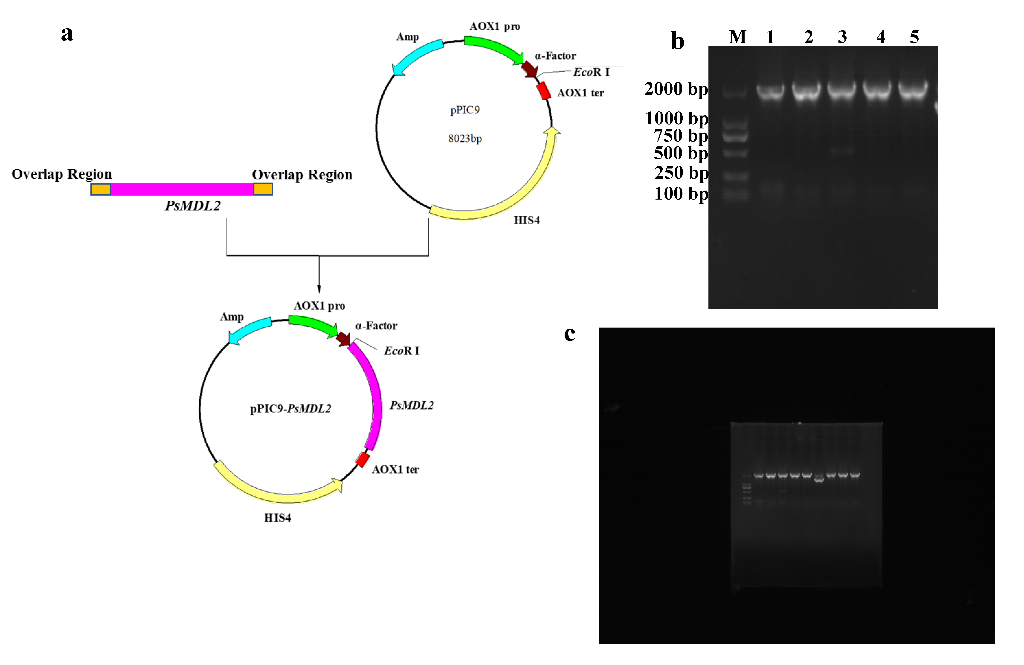
**

**Figure S2.** **The construction of yeast expression vector and identification of the transformed *Pichia pastoris* with recombinant plasmid. (a)** The construction of yeast expression vector of pPIC9/*AOX1::**PsMDL2* applied for PsMDL2 catalytic activity assay. **(b)** Identification of genetically transformed *P. pastoris* with recombinant plasmid by PCR assay. M signified the DNA Marker, and lines 1-5 were the PCR products of DNA from different pPIC9/*AOX1::PsMDL2* colonies. **(c)** The original full-length gel image matched to the cropped version in Figure S2b.
