# Supplementary material for: Mandelonitrile lyase MDL2-mediated regulation of seed amygdalin and oil accumulation of Prunus Sibirica
Source: BMC Plant Biol. 2024 Jun 21;24:590. doi: 10.1186/s12870-024-05300-4 (PMC11191352; doi:10.1186/s12870-024-05300-4)
Supplement: Supplementary file 3 — Supplementary Material 3 [file 12870_2024_5300_MOESM3_ESM.docx]

**Table S1** **Statistical result of** **chi-square test for the screening of T3 homozygotes of transgenic Arabidopsis lines of *PsMDL2* overexpression and mutation**

| Transgenic line | Total | Number of plants | | Ratio | *χ*^2^ | *P* value | Result |
| --- | --- | --- | --- | --- | --- | --- | --- |
|  |  | Resistant plant | Sensitive plant |  |  |  |  |
| *35S::PsMDL2*-1 | 296 | 295 | 1 | 1:0 | 0.003 | 0.956 | Homozygote |
| *35S::PsMDL2*-2 | 304 | 302 | 2 | 1:0 | 0.013 | 0.909 | Homozygote |
| *35S::PsMDL2*-3 | 297 | 297 | 0 | 1:0 | 0 | ＞0.99 | Homozygote |
| *35S::PsMDL2*-4 | 276 | 197 | 79 | 3:1 | 1.932 | 0.165 | Heterozygote |
| *35S::PsMDL2*-5 | 301 | 301 | 0 | 1:0 | 0 | ＞0.99 | Homozygote |
| *mdl2-*1 | 311 | 310 | 1 | 1:0 | 0.003 | 0.98-0.95 | Homozygote |
| *mdl2*-2 | 308 | 308 | 0 | 1:0 | 0 | ＞0.99 | Homozygote |
| *mdl2*-3 | 314 | 314 | 0 | 1:0 | 0 | ＞0.99 | Homozygote |
| *mdl2*-4 | 299 | 298 | 1 | 1:0 | 0.003 | 0.956 | Homozygote |
| *mdl2*-5 | 361 | 283 | 78 | 3:1 | 2.22 | 0.20-0.10 | Heterozygote |
