# Supplementary material for: Mandelonitrile lyase MDL2-mediated regulation of seed amygdalin and oil accumulation of Prunus Sibirica
Source: BMC Plant Biol. 2024 Jun 21;24:590. doi: 10.1186/s12870-024-05300-4 (PMC11191352; doi:10.1186/s12870-024-05300-4)
Supplement: Supplementary file 4 — Supplementary Material 4 [file 12870_2024_5300_MOESM4_ESM.docx]

**
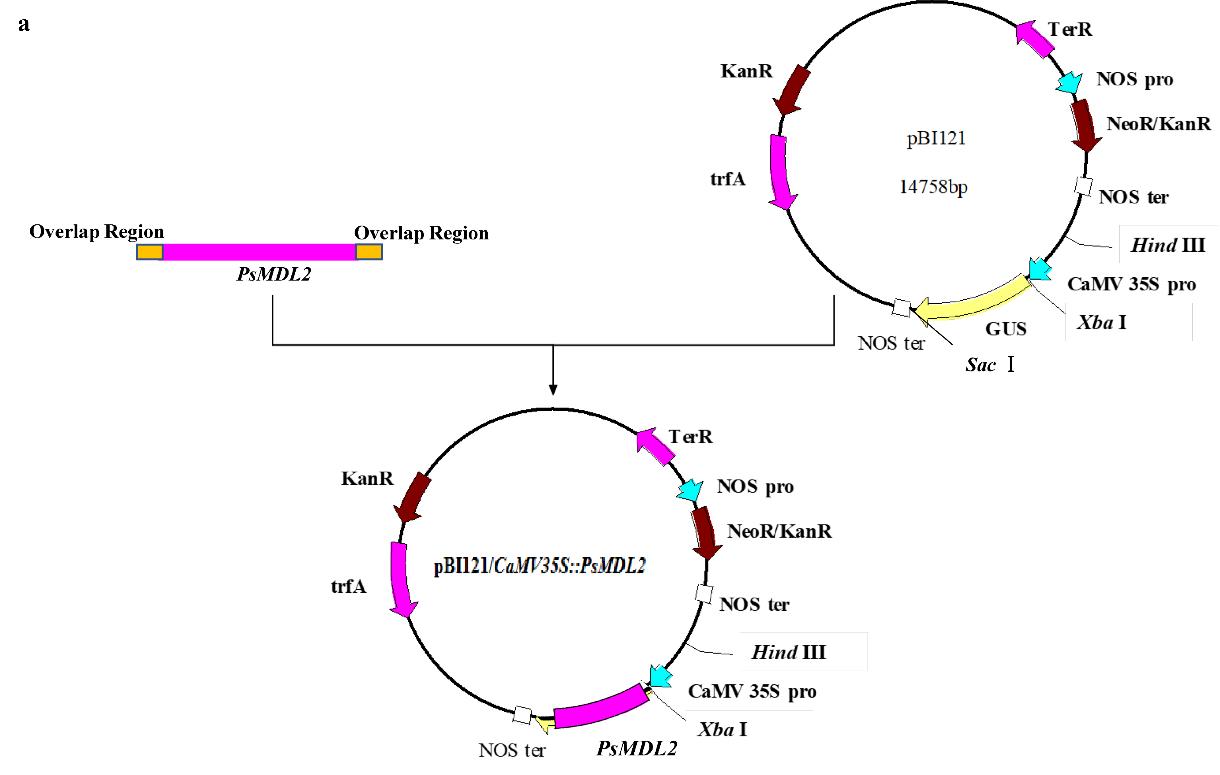
**

**
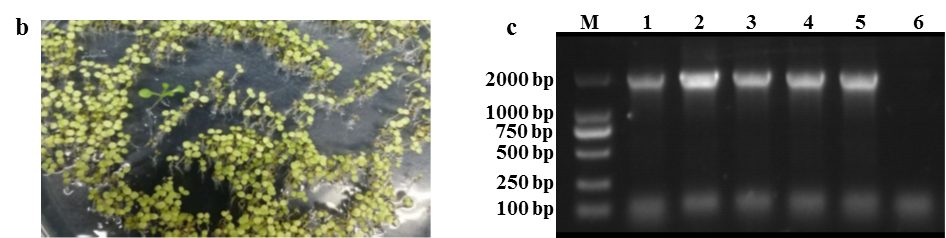
**

**
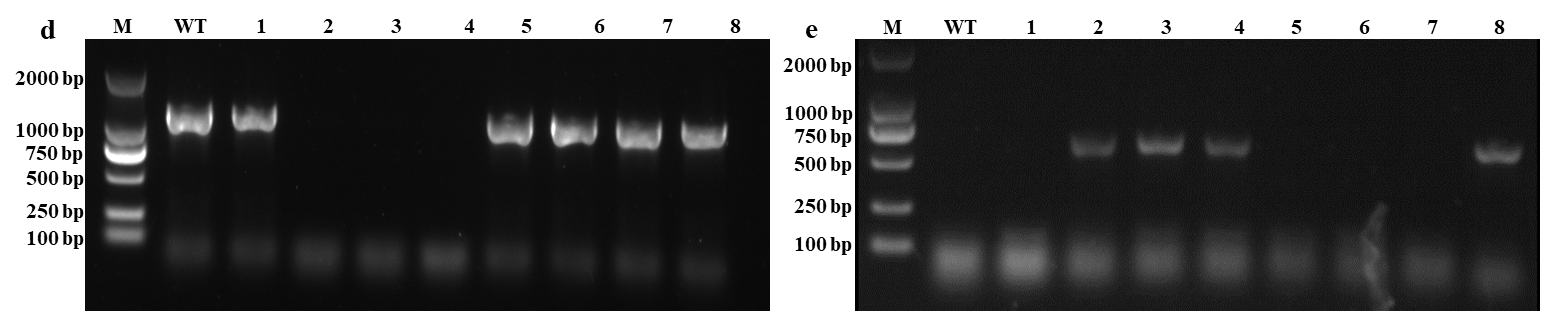
**

**
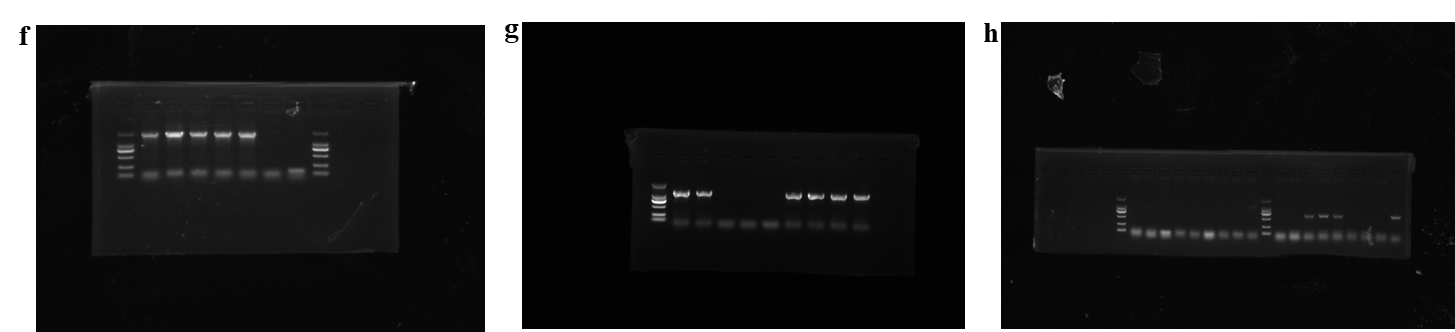
**

**Figure S3.** **The Identifications for homozygous *PsMDL2*-overexpressing plants and *mdl2* mutant lines of Arabidopsis. (a)** Construction of plant expression vector of pBI121/*CaMV35S::PsMDL2* used for transgenic Arabidopsis. **(b)** Screening of Kan resistance for transgenic Arabidopsis with *PsMDL2* overexpression. **(c)** PCR detection for the homozygous plants of transgenic Arabidopsis with *PsMDL2* overexpression. M represented DNA marker, lines 1-5 were homozygous plants of transgenic Arabidopsis, and line 6 was identified as the WT. **(d)** PCR amplification using both LP and RP primers. **(e)** PCR amplification using both BP and RP primers. M represented DNA marker, WT was used as the control, lines 5-7 was identified as the WT, line 8 was heterozygous mutants, and the others were homozygous mutants. **(f)** The original full-length gel image matched to the cropped version in Figure S3c. **(g)** The original full-length gel image matched to the cropped version in Figure S3d. **(h)** The original full-length gel image matched to the cropped version in Figure S3e.
