# Supplementary material for: Mandelonitrile lyase MDL2-mediated regulation of seed amygdalin and oil accumulation of Prunus Sibirica
Source: BMC Plant Biol. 2024 Jun 21;24:590. doi: 10.1186/s12870-024-05300-4 (PMC11191352; doi:10.1186/s12870-024-05300-4)
Supplement: Supplementary file 7 — Supplementary Material 7 [file 12870_2024_5300_MOESM7_ESM.docx]

**
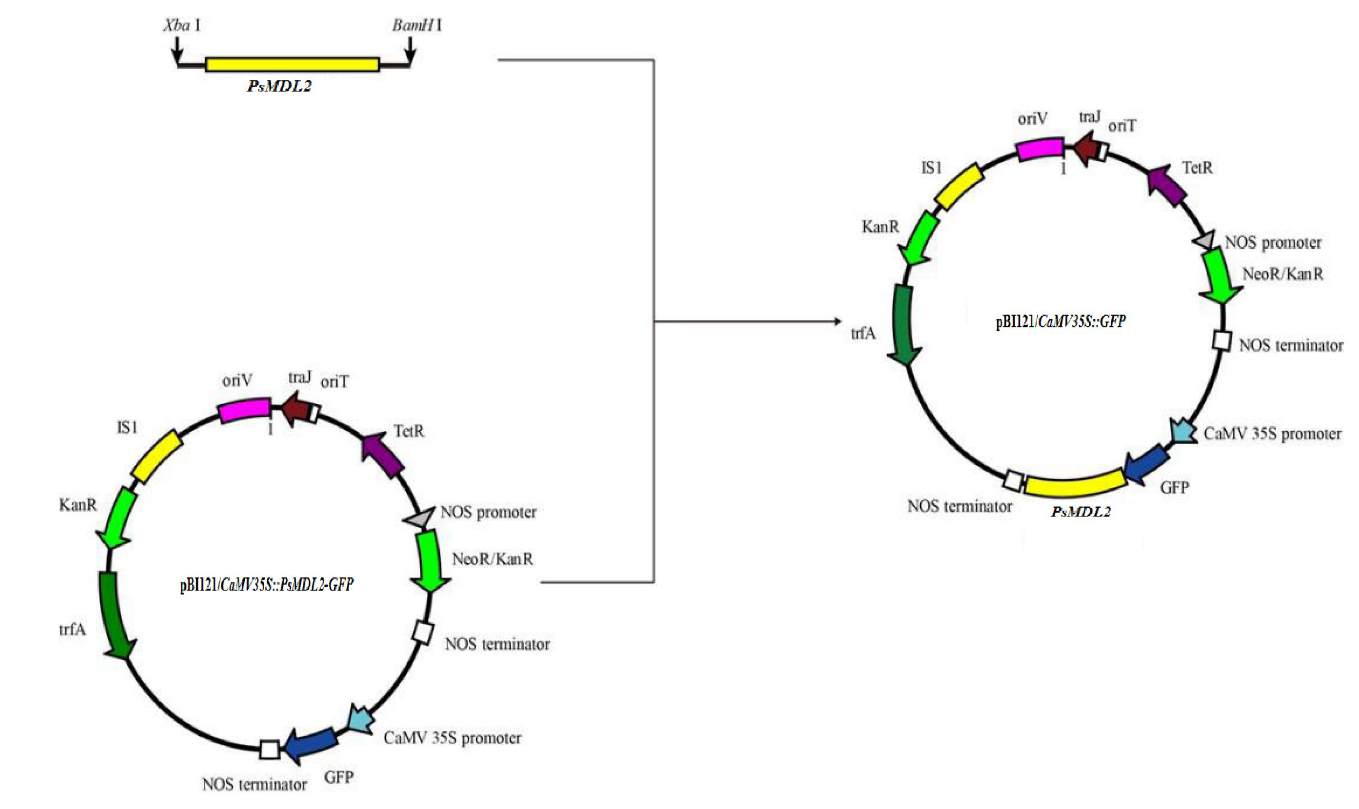
**

**Figure S4.** **Schematic diagram of plant expression vector construction of** **pBI121/*CaMV35S::PsMDL2-GFP*** used for subcellular localization analysis**.**
